# Canopy height and biomass distribution across the forests of Iberian Peninsula

**DOI:** 10.1038/s41597-025-05021-9

**Published:** 2025-04-22

**Authors:** Yang Su, Martin Schwartz, Ibrahim Fayad, Mariano García, Miguel A. Zavala, Julián Tijerín-Triviño, Julen Astigarraga, Verónica Cruz-Alonso, Siyu Liu, Xianglin Zhang, Songchao Chen, François Ritter, Nikola Besic, Alexandre d’Aspremont, Philippe Ciais

**Affiliations:** 1 Département d’Informatique, École Normale Supérieure – PSL, 45 Rue d’Ulm, 75005 Paris, France; 2 Laboratoire des Sciences du Climat et de l’Environnement, CEA CNRS UVSQ Orme des Merisiers, 91190 Gif-sur-Yvette, France; 3 UMR ECOSYS, INRAE AgroParisTech, Université Paris-Saclay, 91120 Palaiseau, France; 4 Universidad de Alcalá, Departamento de Geología, Geografía y Medio Ambiente, Environmental Remote Sensing Research Group, 28801 Alcalá de Henares, Spain; 5 Universidad de Alcalá, Departamento de Ciencias de la Vida, Forest Ecology and Restoration Group (FORECO), 28805 Alcalá de Henares, Spain; 6 Department of Biodiversity, Ecology and Evolution, Complutense University of Madrid, 28040 Madrid, Spain; 7 Department of Geosciences and Natural Resource Management, Copenhagen University, 1958 Frederiksberg, Denmark; 8 College of Environmental and Resource Sciences, Zhejiang University, 310058 Hangzhou, China; 9 ZJU-Hangzhou Global Scientific and Technological Innovation Center, Zhejiang University, 311215 Hangzhou, China; 10 IGN, ENSG, Laboratoire d’inventaire forestier (LIF), 54000 Nancy, France

**Keywords:** Forestry, Forestry

## Abstract

Accurate mapping of vegetation canopy height and biomass distribution is essential for effective forest monitoring, climate change mitigation, and sustainable forestry. Here we present high-resolution remote sensing-based canopy height (10 m resolution) and above ground biomass (AGB, 50 m resolution) maps for the forests of the Iberian Peninsula from 2017 to 2021, using a deep learning framework that integrates Sentinel-1, Sentinel-2, and LiDAR data. Two UNET models were developed: one trained on Airborne Laser Scanning (ALS) data (MAE: 1.22 m), while another using Global Ecosystem Dynamics Investigation (GEDI) footprints (MAE: 3.24 m). External validation with 6,308 Spanish National Forest Inventory (NFI) plots (2017–2019) confirmed canopy height reliability, showing MAEs of 2–3 m in tree-covered areas. AGB estimates were obtained through Random Forest models that linked UNET derived height predictions to NFI AGB data, achieves an MAE of ~29 Mg/ha. The creation of high-resolution maps of canopy height and biomass across various forest landscapes in the Iberian Peninsula provides a valuable new tool for environmental researchers, policy makers, and forest management professionals, offering detailed insights that can inform conservation strategies, carbon sequestration efforts, and sustainable forest management practices.

## Background & Summary

Forests are essential components of the Earth’s biosphere, providing a range of ecosystem services, including carbon sequestration, biodiversity reserving, and human livelihoods supporting. Accurate assessment of forest structure, specifically canopy height and biomass, is crucial for understanding the role of forests in carbon cycling, climate regulation, wood resources and ecosystem dynamics^1,2^. Recent advancements in remote sensing and deep learning technologies have significantly enhanced our capacity to monitor forest structure at both regional and global scales^3–7^.

Satellite instruments like Sentinel-1 (S1) and Sentinel-2 (S2) provide high-resolution, synthetic aperture radar (SAR) and optical multispectral images, enabling extensive observation of land areas with high temporal frequency^8,9^. Meanwhile, forest height and canopy vertical/horizontal structure have been monitored globally since 2018 by the Global Ecosystem Dynamics Investigation (GEDI) mission at discrete locations (25 m diameter footprints) between 51.6°N and 51.6°S^6^ and by Airborne Laser Scanning (ALS) campaigns which offer high accurate data but are limited by infrequent operations and narrow spatial coverage^10^.

The GEDI high energy lidar instrument, aboard the International Space Station, executes laser ranging measurements essential for mapping global forest canopy heights and directly assessing their vertical structures. Although it achieves extensive coverage across large geographic expanses, the configuration of its measurements, with a spacing of 60 m between footprints along each of the four parallel laser tracks and 600 m between tracks, results in an incomplete spatial coverage given the high heterogeneity of forests^11–14^. In contrast, the ALS campaigns use laser instrument mounted on aircraft to continuously measure the terrain with higher spatial resolution, ideal for in-depth studies at a local scale but at a higher cost and logistical complexity than satellite observations. Merging these canopy height reference datasets with satellite imagery enables the development of predictive models of forest structure^15,16^. The introduction of deep learning techniques, especially Convolutional Neural Networks (CNNs) like UNET^17,18^, has revolutionized the field of remote sensing. These technologies enable the detection of intricate patterns within satellite images, significantly enhancing the precision of environmental mapping^19^.

Satellite-based global forest height maps, created using Random Forest (RF) and conventional CNNs, have been produced in years such as 2019^5^ and 2020^20^. Yet, these global models often lack regional precision and struggle to accurately track forest disturbances. To address these limitations, we developed two deep learning models based on the UNET architecture. Designed specifically for the precise mapping of canopy height across the forests of the Iberian Peninsula at 10 m resolution, these two models utilize satellite imagery from S1 and S2 spanning 2017 to 2021. Each model is trained by distinct target height data, one by the GEDI dataset and the other by the ALS dataset. The results from both models were cross-compared and also independently validated against the *in-situ* canopy height records from the fourth census of the Spanish National Forest Inventory (NFI) for the years 2017 to 2019. This validation highlights the effectiveness of our methodology in bridging the existing gap in forest height mapping and disturbance tracking in the Iberian Peninsula.

After the prediction of canopy height, we trained RF^21,22^ models, based on the integration of predicted canopy height data with NFI above-ground biomass (AGB) records (the NFI plot has a diameter of 50 m) to construct yearly AGB maps at 50 m resolution^23^. By generating high-resolution maps of canopy height (10 m resolution) and maps of AGB (50 m resolution), this study provides valuable insights for forest management, conservation efforts, and climate change mitigation strategies.

## Method

### Data collection

We obtained satellite imagery data through Google Earth Engine (GEE)^24^, sourcing S1 data from the collection “COPERNICUS/S1_GRD”^25^ and S2 data from “COPERNICUS/S2_SR”^26^. The S1 dataset includes VV and VH bands, captured by Sentinel-1 satellites equipped with C-band SAR, which provides valuable information on forest structure that complements the data from passive-optical sensors. It is essential for tasks like forest surveillance^27^. In the S1 data, VV polarization refers to signals transmitted and received in a vertical polarization, while VH polarization represents signals transmitted in vertical but received in horizontal polarization. The S2 dataset obtained from level-2A product of Sentinel-2 satellites, includes B2, B3, B4, B5, B6, B7, B8, B8A, B11, B12, TCI_R, TCI_G and TCI_B bands. S2 dataset provides multispectral images across 13 spectral bands and is processed into surface reflectance (SR) format to minimize atmospheric distortions and accurately represent Earth’s surface. These data are particularly valuable for detailed monitoring of vegetation and land cover, supporting precise environmental management and land use analysis^28^. The S1 and S2 datasets offer a resolution up to 10 m and cover the period from 2017 to 2021. We downloaded the annual median images from both S1 and S2 with the coordinate reference system (CRS) of EPSG: 3042. Since S2 data is susceptible to cloud interference, we implemented a cloud filter based on QA60 band to enhance image quality. The detailed filter setting can be found in Supplementary Table 1. While most S2 bands are at 10 m resolution, B5, B6, B7, and B8A originally have a 20 m resolution but were resampled to 10 m by assigning the same values to a finer grid. The code for downloading the S1 and S2 data are available via GEE scripts (https://code.earthengine.google.com/8ec80583b3bcc7c1654bb83d657bf0a2) and (https://code.earthengine.google.com/70a5ce97ccbc9d4c61bf08047999298f), respectively.

From the ALS data, a canopy height model (CHM) was generated by subtracting the Digital Terrain Model (DTM) from the Digital Surface Model (DSM) using the following formula:

$${CHM}={DSM}-{DTM}$$

The DTM and DSM were generated from the second comprehensive LiDAR data coverage of Spain conducted between 2015 and 2022^29^. The data are publicly accessible via PNOA LiDAR Data website^30^. For our analysis, we used the data spanning from 2017 to 2021, covering 205,213 km^2^ of Spain or 35.18% of the area of the Iberian Peninsula. This coverage spans a diverse range of land covers and land uses (Supplementary Figure 1a). The original ALS data have an average pulse density of 1 pulse per m^2^, with values ranging from 0.5 to 14 pulses per m², depending on the region. The LiDAR returns are filtered and made available in LAZ format. From this data, the DTM and DSM were generated at 2 m resolution. After constructing the CHM, we resampled it into a 10 m resolution map by selecting the maximum value from the 2 m resolution pixels, which represent the maximum canopy height within each 10 m resolution pixel. For more detailed information about the flight dates, LiDAR flight characteristics, sensor types, surveyed regions, accuracy, and other relevant details, please consult the PNOA LiDAR General Information webpage^31^. Technical specifications for this LiDAR coverage are available on the PNOA LiDAR Technical Specifications webpage^32^. Note that, the relatively low pulse density of ALS (0.5–14 pulses per square meter) may occasionally miss the highest portions of the canopy, potentially resulting in underestimations of maximum canopy height. Nevertheless, ALS data offer substantially high spatial resolution, delivering detailed structural information and facilitating accurate, spatially explicit canopy height mapping.

Regarding the GEDI canopy height dataset, it was retrieved via GEE from the collection labeled “LARSE/GEDI/GEDI02_A_002_MONTHLY”^6,33^, which is GEDI’s Level 2 A Geolocated Elevation and Height Metrics Product. This dataset encompasses the entire Iberian Peninsula (Supplementary Figure 1b), with data available from 2019 onwards. We downloaded the data from 2019 to 2021 and used the RH98 value to represent the maximum canopy height. To ensure the best quality of data, we applied a series of meticulous filters. In addition to the filters that were used in a previous study^18^, to eliminate inaccuracies caused by surface reflectance from sunlight, we included data collected only during nighttime, defined as periods when the solar elevation was below zero. To minimize the high uncertainty associated with GEDI measurements in steep terrain^34^, we limited the inclusion of GEDI data to the regions with a slope of no more than 10 degrees. Furthermore, we avoided data points within 25 m of forest edges based on the focal.max() operator on GEE and “ESA/WorldCover/v200” tree cover class^35^ to prevent geolocation errors^36^. Moreover, to only include the vegetation into consideration, we adjusted the GEDI RH98 measurements during the data preparation stage to the ground-return heights for areas classified as permanent water bodies or built-up areas according to the “ESA/WorldCover/v200” dataset^35^. During post-processing, we further set the predicted height to 0 m for areas identified as ground by the model. We obtained 2,579,421 footprints after data filtering, with the detailed filter settings provided in Supplementary Table 1. While the native resolution of the GEDI dataset stands at 25 m, we rescaled it to the resolution of 10 m for our download by distributing the original data across smaller 10-meter pixels. The code for downloading GEDI canopy height data is available via GEE (https://code.earthengine.google.com/7f9dc05850ecd689aa2c8fc1a49e4597).

As for the GEDI tree coverage ratio dataset, it was obtained via GEE from the collection named “LARSE/GEDI/GEDI02_B_002_MONTHLY”, which corresponds to GEDI’s Level 2B Raster Canopy Cover Vertical Profile Metrics (Version 2). This dataset shares the same spatial and temporal extent as the GEDI canopy height dataset. We extracted the total canopy cover (range from 0 to 1) from this dataset using quality filters similar to those applied to the GEDI canopy height dataset, as detailed in Supplementary Table 1. The script used for downloading the GEDI tree coverage ratio data is accessible via GEE script (https://code.earthengine.google.com/7b79f46e593e236957f0f638ff0ab17d).

The NFI data were sourced from the ongoing 4^th^ census of the Spanish NFI^37–39^, comprising 57723 plots so far with a tree cover greater than 5% laid out on a 1 km² grid. The plots follow a concentric circular design with a varying size plot sampling method depending on tree size (diameter at breast height or DBH). The full plot encompasses a circle with a total diameter of 50 m^37–39^, and the latitude and longitude of the center point is recorded. The radii of these concentric plots vary according to the tree’s DBH: a 25 m radius for DBH ≥ 42.5 cm, a 15 m radius for 42.5 > DBH ≥ 22.5 cm, a 10 m radius for 22.5 > DBH ≥ 12.5 cm, and a 5 m radius for 12.5 > DBH ≥ 7.5 cm. For all trees measured within their respective radii, height is recorded from the base of the trunk to the apex of the crown. These height measurements allow for the calculation of different height metrics, including maximum height. Maximum height is the maximum value of the sampled trees according to this procedure. This methodology ensures a comprehensive and standardized assessment of forest structure across the Spanish territory. Among representative plot-level height metrics (maximum height, dominant height, mean height, or Lorey’s height), maximum height is generally the most resilient to missing measurements. And for this study only data from the adult trees was used (height >130 cm and DBH >7.5 cm).

Spanning from 2008 to 2019, this NFI dataset study covers a large area of Spain (Supplementary Figure 1c). From this dataset, precise geolocation, census year, maximum canopy height, and above-ground biomass were extracted. Plots where all trees were recorded as dead or where only one individual tree was present were excluded, leaving 16,566 permanent plots for the analysis. The AGB was estimated using species-specific allometric equations based on tree DBH^40^ and canopy height. During the external validation, the maximum canopy height data was corrected for bias using observations from ALS to correct systematic discrepancies between human and aerial measurements and to address data gaps that arise from disturbances like fires occurring within the year of the visit (details available in **Supplementary document**). Due to the mission launch constraints in the S1 and S2 data, we selected a subset of 6308 plots from the years 2017 to 2019 (Supplementary Figure 1d) to serve as the external validation set for our canopy height models and as training and testing data for the biomass models.

### Model description

Originally designed for segmenting biomedical images, the UNET model^17^, has been successfully adapted for remote sensing tasks, thanks to its specialized architecture for detailed image segmentation. Characterized by its distinctive U-shaped structure (Supplementary Figure 2), the UNET architecture facilitates the analysis of images at different scales, effectively capturing the spatial hierarchy and the relationship between adjacent pixels, even with limited data. This enables UNET to effectively identify and segment complex land cover features such as forests, croplands, grasslands, human-built environments and lakes^19^. This feature is particularly valuable for detecting canopy heights in various types of landscapes at the country level^18^.

### Model training, validation and testing

**Fig. 1:**
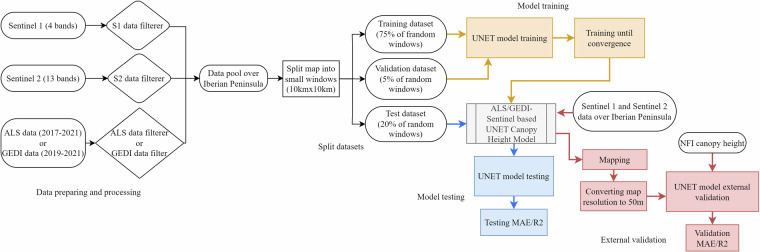
Flow chart of training, testing and validation of UNET canopy height models. This plot showed the algorithm of UNET canopy height model training (marked by orange color), testing (marked by blue color) and external validation (marked by pink color) for ALS based UNET model and the GEDI based UNET model, respectively, depending on the input data.

In this study, we trained UNET canopy height models^17,18^ with the discrete point measurements of canopy height to use S1 and S2 satellite imagery for continuous spatio-temporal mapping of canopy heights. Figure 1 outlines the procedure for training, validating, and testing our canopy height models. The detailed model structure and model parameters can be found in Supplementary Tables 3, 4. We trained two UNET models utilizing different sources of height reference data: ALS or GEDI canopy height data (Fig. 1). While the structure of both models remains identical, the difference lies in the reference data employed. GEDI data provide extensive spatial coverage, yet it does not provide spatially continuous height measurements due to its configuration and spanning from 2019 to 2021. Conversely, ALS data offer smaller spatial coverage, but with higher spatial resolution, and spanning from 2017 to 2021.

We integrated satellite imagery with height reference data from ALS and GEDI, aligning them based on precise geographic locations and the census year, respectively, to create two comprehensive canopy height datasets. Each dataset was subsequently divided into smaller 10 km by 10 km segments (or 1000 pixels by 1000 pixels), amounting to 13,100 small windows per year across the entire Iberian Peninsula. These windows were then randomly split into 3 parts, 75% were assigned to the training set, 5% were utilized to the validation set for monitoring the training progress of the UNET models (with training stopping once the validation loss converges), and the remaining 20% were reserved as the out-of-box testing set to evaluate the final models’ performances. During the training phase of the model, we processed 12 windows at a time. From each 1000 × 1000 pixel window, a sub-window of 256 × 256 pixels is randomly extracted. This technique of random cropping is implemented to enhance the model’s exposure to diverse data scenarios^41^, improve its ability to generalize across different spatial features, and increase the model robustness^18^. It should be noted that windows lacking canopy height reference data will be automatically excluded by the model, meaning they will not be included in loss calculations. Following the training and testing of both the ALS-Sentinel based UNET model and the GEDI-Sentinel based UNET model, we conducted the external validation with the NFI maximum canopy height observations. To do so, we first generated canopy height maps for the period from 2017 to 2021 using annual inputs from S1 and S2 satellites during the same timeframe. The accuracy of the canopy height maps produced by the GEDI UNET model for 2017 and 2018 is lower due to the absence of reference data from GEDI for those years. Considering the NFI plot size (50 m-diameter), we extracted canopy height values by first locating each NFI observation point within our maps. For each observation, we searched within a 50 m-diameter circular area and selected the maximum canopy height value within that region. This maximum value was then compared with the corresponding NFI observation recorded at the same geolocation and year of visit to conduct an external validation of our model’s accuracy. The models’ effectiveness was assessed using the following metrics: MAE, rooted mean squared error (RMSE) and R^2^.

To prepare the data for AGB prediction, we followed a similar approach as the training of the UNET canopy height model to train a UNET model specifically for tree coverage ratio based on GEDI canopy cover data (Supplementary Figure 3). Those data were also randomly split into 3 parts, 75% for training, 5% for validation, and 20% for testing. Subsequently, we produced the tree coverage ratio maps at 10 m resolution spanning from 2017 to 2021, then converted to 50 m resolution by taking the average value of the 10 m resolution pixels inside the 50 m resolution pixel.

To predict AGB, we developed RF models^21,22^ to analyze independent AGB data sourced from the NFI, alongside canopy maximum heights derived from the UNET canopy height models and tree coverage ratios from the UNET tree coverage ratio model. Canopy height is closely related to AGB because taller trees typically store greater biomass, as tree height is strongly correlated with stem volume and wood mass^2^. Similarly, the tree coverage ratio indicates the proportion of land area (within each 50 m resolution pixel) occupied by tree canopies, providing essential context for biomass estimates, as higher canopy coverage generally correlates with increased biomass accumulation^3,42^. Additionally, based on the results of one-way ANOVA, significant variances were observed in both latitude and longitude across forests dominated by tree species with different leaf types (e.g., evergreen needleleaf, evergreen broadleaf, and deciduous broadleaf), with latitude displaying more pronounced differences (as depicted in Supplementary Figure 4). Consequently, we incorporated latitude and longitude data in the RF models to approximate forest type information and assess the influences of climate, enhancing our model’s capacity to predict variations in biomass influenced by geographic location and the distribution of different forest types. Compared with the method that calculates the tree allometry function to predict biomass from canopy height^18^, this methodology exploits the capacity of RF model in handling complex, non-linear relationships within the data^22,43^ and benefits from the precision of UNET in capturing canopy heights and tree coverage ratio.

**Fig. 2:**
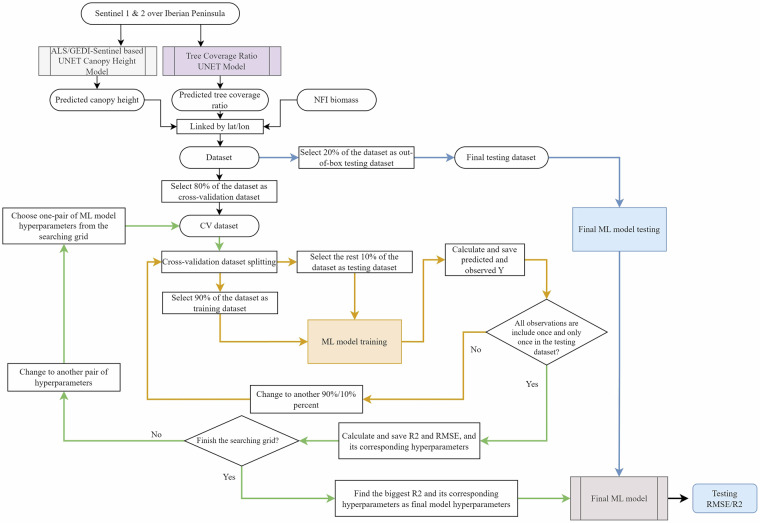
Flow chart of training, tuning with 10-fold cross validation and testing of AGB models. This plot showed the algorithm of RF AGB model training, tuning with 10-fold cross-validation (marked by orange color) and grid searching (marked by green color), final testing (marked by blue color) with the input data of predicted canopy height, tree coverage ratio and geocoordinates.

The detailed procedure for AGB model training, tuning with cross-validation, and testing are illustrated in Fig. 2. We first extracted the canopy heights from the down-sampled 50 m resolution maps produced by UNET canopy height model, and the tree coverage ratio from the down-sampled 50 m resolution maps produced by UNET tree coverage ratio model, at the locations and the census year of the NFI records. This extraction allowed us to assemble a dataset incorporating the predicted canopy heights, predicted tree coverage ratio, latitude, longitude and the AGB data from NFI. In the original NFI dataset, the AGB values were missing for non-vegetated areas. To address this, we randomly selected a subset of bare soil, water bodies, and built-up areas (locations identified as ground by the model) equivalent to 10% of the total NFI dataset size. These selected locations were assigned an AGB of 0 and included in the training dataset. Incorporating these zero-AGB data points helps fill data gaps and ensures more accurate and unbiased AGB estimates, especially for areas with little or no vegetation. Subsequently, we divided this dataset into two portions: 80% as the cross-validation dataset for model training and parameter tuning, and the remaining 20% as out-of-box testing dataset.

For model tuning, we employed a grid search strategy^43,44^ with 10-fold cross-validation^45^ to optimize the hyperparameters, specifically focusing on the number of features randomly selected for building each tree (“mtry” in the following), and the total number of trees in the forest (“ntree” in the following)^21,22^. We varied “mtry” from 1 to 4 and “ntree” from 300 to 1000. For each combination of these hyperparameters, we performed a 10-fold cross-validation to evaluate the model using R^2^ and RMSE metrics, systematically recording results from each iteration across the grid. The combination yielding the lowest RMSE was chosen as the final set of hyperparameters.

Finally, we tested the model using the out-of-box testing dataset to assess the overall model performance. Given that we utilized two separate UNET models for predicting canopy heights, we correspondingly developed two distinct RF models (specifically, ALS-Sentinel based RF model and GEDI-Sentinel based RF model) to integrate the varying canopy height data provided by ALS-Sentinel based and GEDI-Sentinel based UNET canopy height models.

## Data Records

We have made available 27 image collections on Zenodo^46–63^, providing high-resolution maps of canopy height, tree coverage ratio, above-ground biomass, and forest disturbances for the Iberian Peninsula. These datasets span from 2017 to 2021 and are derived from multiple remote sensing sources, including ALS, GEDI, Sentinel-1, and Sentinel-2 imagery.

### Dataset overview

**Fig. 3:**
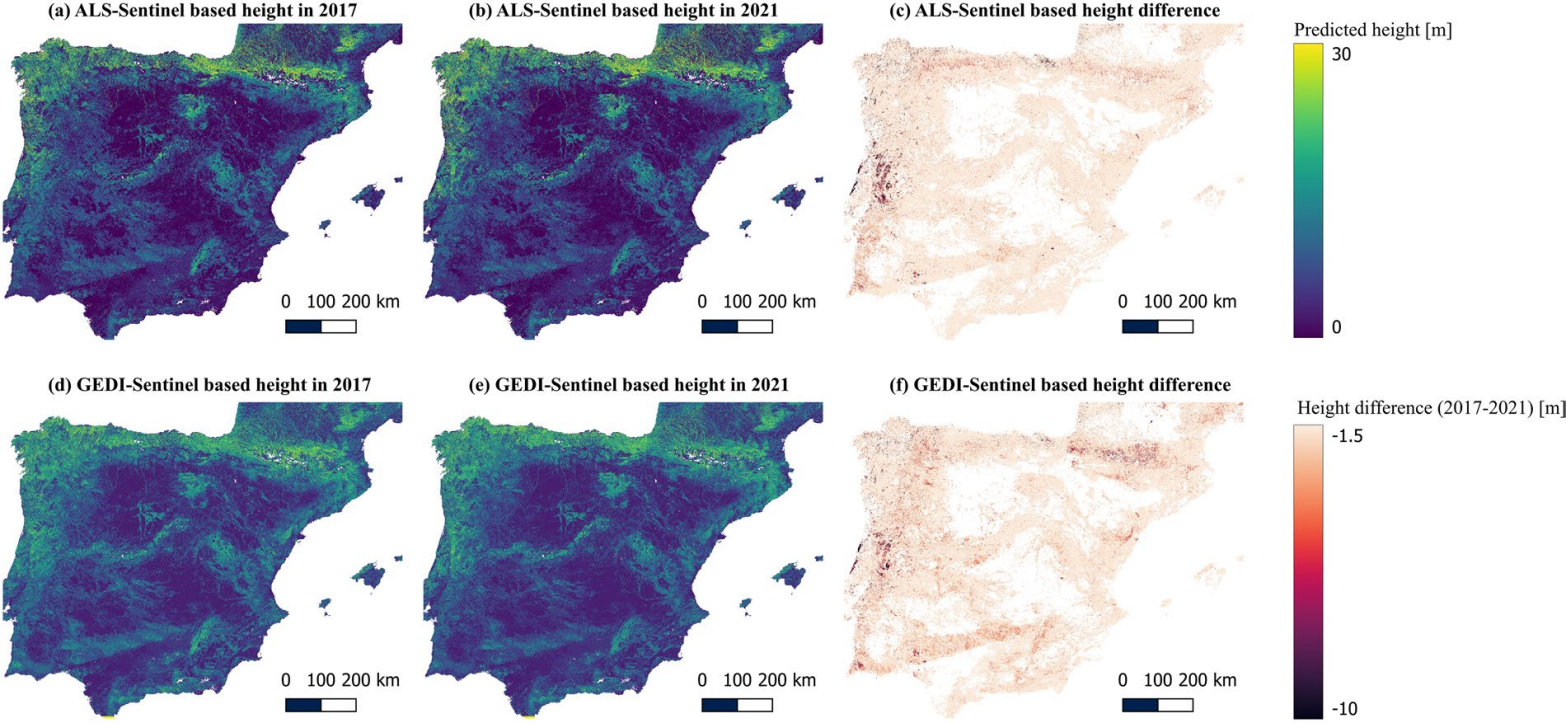
Canopy height in the year 2017 and 2021. Plot a and b are the canopy heights in 2017 and 2021 predicted by the ALS-Sentinel based UNET canopy height model, respectively. Plot d and e are the canopy heights in 2017 and 2021 predicted by the GEDI-Sentinel based UNET canopy height model, respectively. Plot c and f are the height difference predicted by the ALS-Sentinel based and GEDI-Sentinel based UNET canopy height models, respectively.
- Tree Coverage Ratio Maps (5 maps)
  - Derived from the UNET-based tree coverage ratio model trained with GEDI total canopy cover data.
  - Resolution: 10 m | CRS: EPSG:3042 | Unit: Fraction (0–1).
  - Visualized in Supplementary Figure 5a,b.
- Above Ground Biomass Maps (10 maps)
  - Produced by ALS-Sentinel based RF model: Five yearly maps (2017–2021).
  - Produced by GEDI-Sentinel based RF model: Five yearly maps (2017–2021).
  - Resolution: 50 m | CRS: EPSG:3042 | Unit: Mg/ha).
  - Visualized in Fig. 4a,b (ALS-Sentinel) and Fig. 4d,e (GEDI-Sentinel).

This collection includes ten canopy height maps, five tree coverage ratio maps, and ten above-ground biomass maps. More specifically, the dataset features:

- Canopy Height Maps (10 maps)
  - Produced by ALS-Sentinel based UNET model: Five yearly maps (2017–2021).
  - Produced by GEDI-Sentinel based UNET model: Five yearly maps (2017–2021).
  - Resolution: 10 m | CRS: EPSG:3042 | Unit: meters.
  - Visualized in Fig. 3a,b (ALS-Sentinel) and Fig. 3d,e (GEDI-Sentinel).

**Fig. 4:**
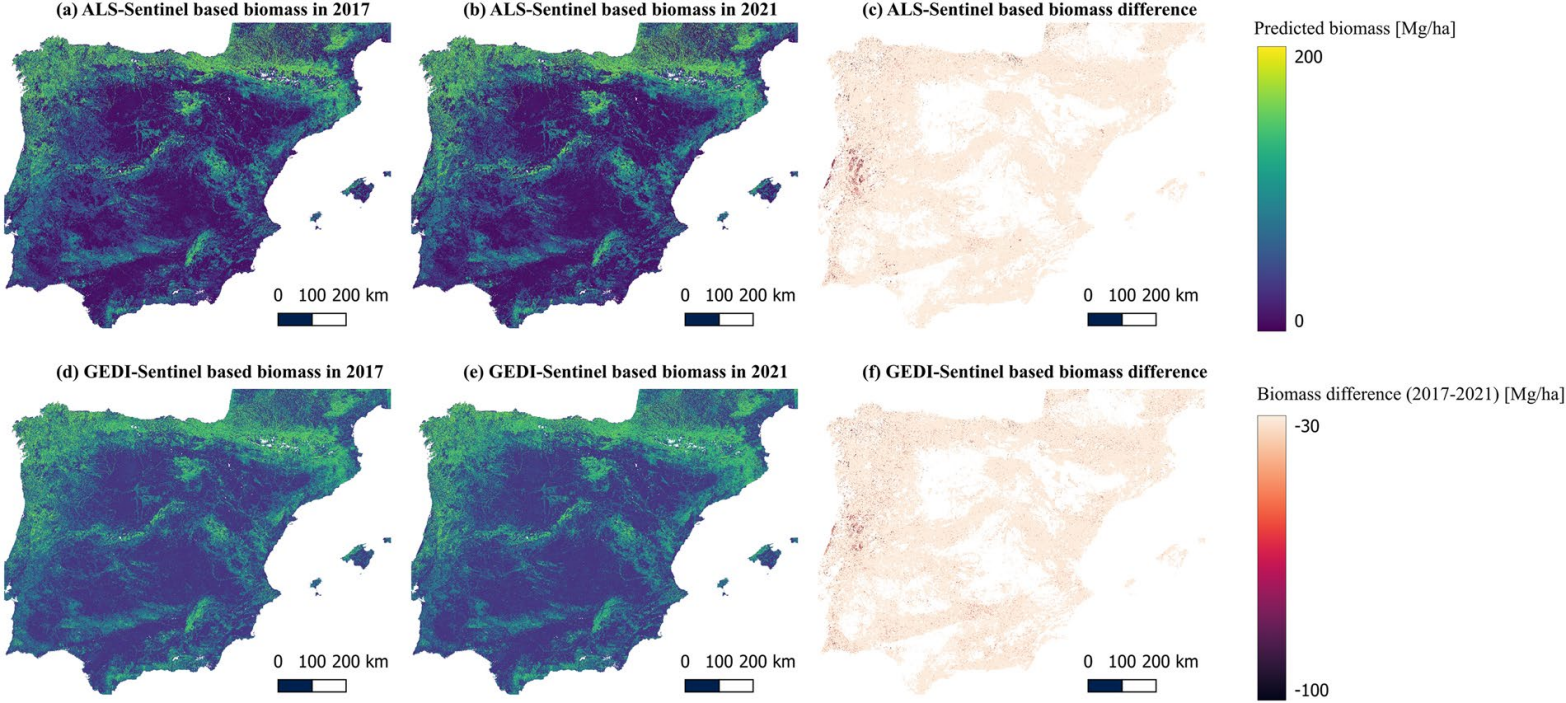
AGB in the year 2017 and 2021. Plot a and b are the biomass in 2017 and 2021 predicted by the ALS-Sentinel based RF model, respectively. Plot d and e are the biomass in 2017 and 2021 predicted by the GEDI-Sentinel based RF model, respectively. Plot c and f are the biomass difference predicted by the ALS-Sentinel based and GEDI-Sentinel based UNET model, respectively.
- Forest Disturbance Maps (2 maps)
  - Derived from ALS-Sentinel and GEDI-Sentinel based canopy height change between 2017 and 2021.
  - Resolution: 10 m | CRS: EPSG:3042 | Unit: meters).
  - Visualized in Fig. 3c,f.
  - These maps help quantify areas affected by deforestation, degradation.

These maps are publicly accessible via Zenodo and can be downloaded through the URLs provided in Tables 1–5. Each dataset is stored in GeoTIFF format, ensuring compatibility with standard GIS software and remote sensing tools. The canopy height maps, and tree coverage ratio maps are available at a resolution of 10 m, whereas the biomass maps have been prepared at a resolution of 50 m. For a direct visualization of these maps and other related geographic layers, including disturbance maps, and disturbance size maps, please visit: https://ens-yangsu-forest-spain-als.projects.earthengine.app/view/ai4forest-iberian-peninsula.

**Table 1:** Zenodo repository link to ALS-Sentinel based canopy height data.

Seq	Data name	Link to Zenodo assets	Resolution	Unit	Spatial coverage	File format	CRS	Size
1	Canopy height map in 2017 based on ALS-Sentinel UNET model	10.5281/zenodo.14968747	10 m	meters	Iberian Peninsula	Geotiff	3042	45.09GB
2	Canopy height map in 2018 based on ALS-Sentinel UNET model	10.5281/zenodo.14975911	10 m	meters	Iberian Peninsula	Geotiff	3042	45.46GB
3	Canopy height map in 2019 based on ALS-Sentinel UNET model	10.5281/zenodo.14977001	10 m	meters	Iberian Peninsula	Geotiff	3042	45.52GB
4	Canopy height map in 2020 based on ALS-Sentinel UNET model	10.5281/zenodo.14977357	10 m	meters	Iberian Peninsula	Geotiff	3042	45.47GB
5	Canopy height map in 2021 based on ALS-Sentinel UNET model	10.5281/zenodo.14977506	10 m	meters	Iberian Peninsula	Geotiff	3042	45.42GB

**Table 2:** Zenodo repository link to GEDI-Sentinel based canopy height data.

Seq	Data name	Link to Zenodo assets	Resolution	Unit	Spatial coverage	File format	CRS	Size
1	Canopy height map in 2017 based on GEDI-Sentinel UNET model	10.5281/zenodo.14977626	10 m	meters	Iberian Peninsula	Geotiff	3042	43.21GB
2	Canopy height map in 2018 based on GEDI-Sentinel UNET model	10.5281/zenodo.14977728	10 m	meters	Iberian Peninsula	Geotiff	3042	43.05GB
3	Canopy height map in 2019 based on GEDI-Sentinel UNET model	10.5281/zenodo.14982682	10 m	meters	Iberian Peninsula	Geotiff	3042	43.12GB
4	Canopy height map in 2020 based on GEDI-Sentinel UNET model	10.5281/zenodo.14983197	10 m	meters	Iberian Peninsula	Geotiff	3042	43.15GB
5	Canopy height map in 2021 based on GEDI-Sentinel UNET model	10.5281/zenodo.14983837	10 m	meters	Iberian Peninsula	Geotiff	3042	42.99GB

**Table 3:** Zenodo repository link to ALS-Sentinel based and GEDI-Sentinel based above-ground biomass data.

Seq	Data name	Link to Zenodo assets	Resolution	Unit	Spatial coverage	File format	CRS	Size
1	Biomass map in 2017-2021 from ALS-Sentinel based model (5 maps)	10.5281/zenodo.15032832	50 m	Mg/ha	Iberian Peninsula	Geotiff	3042	6.70GB
2	Biomass map in 2017-2021 from GEDI-Sentinel based model (5 maps)	10.5281/zenodo.15032631	50 m	Mg/ha	Iberian Peninsula	Geotiff	3042	7.51GB

**Table 4:** Zenodo repository link to GEDI-Sentinel based tree coverage ratio.

Seq	Data name	Link to Zenodo assets	Resolution	Unit	Spatial coverage	File format	CRS	Size
1	Tree coverage ratio map in 2017 based on GEDI-Sentinel UNET tree coverage ratio model	10.5281/zenodo.15032307	10 m	0-1 (fractional)	Iberian Peninsula	Geotiff	3042	45.87GB
2	Tree coverage ratio map in 2018 based on GEDI-Sentinel UNET tree coverage ratio model	10.5281/zenodo.15032393	10 m	0-1 (fractional)	Iberian Peninsula	Geotiff	3042	45.78GB
3	Tree coverage ratio map in 2019 based on GEDI-Sentinel UNET tree coverage ratio model	10.5281/zenodo.15032448	10 m	0-1 (fractional)	Iberian Peninsula	Geotiff	3042	45.87GB
4	Tree coverage ratio map in 2020 based on GEDI-Sentinel UNET tree coverage ratio model	10.5281/zenodo.15032488	10 m	0-1 (fractional)	Iberian Peninsula	Geotiff	3042	45.67GB
5	Tree coverage ratio map in 2021 based on GEDI-Sentinel UNET tree coverage ratio model	10.5281/zenodo.15032553	10 m	0-1 (fractional)	Iberian Peninsula	Geotiff	3042	45.74GB

**Table 5:** Zenodo repository link to ALS-Sentinel based and GEDI-Sentinel based disturbance map.

Seq	Data name	Link to Zenodo assets	Resolution	Unit	Spatial coverage	File format	CRS	Size
1	Canopy height derived disturbance from 2017 to 2021 (2 maps)	10.5281/zenodo.15072417	10 m	meters	Iberian Peninsula	Geotiff	3042	187.07MB

## Technical Validation

**Fig. 5:**
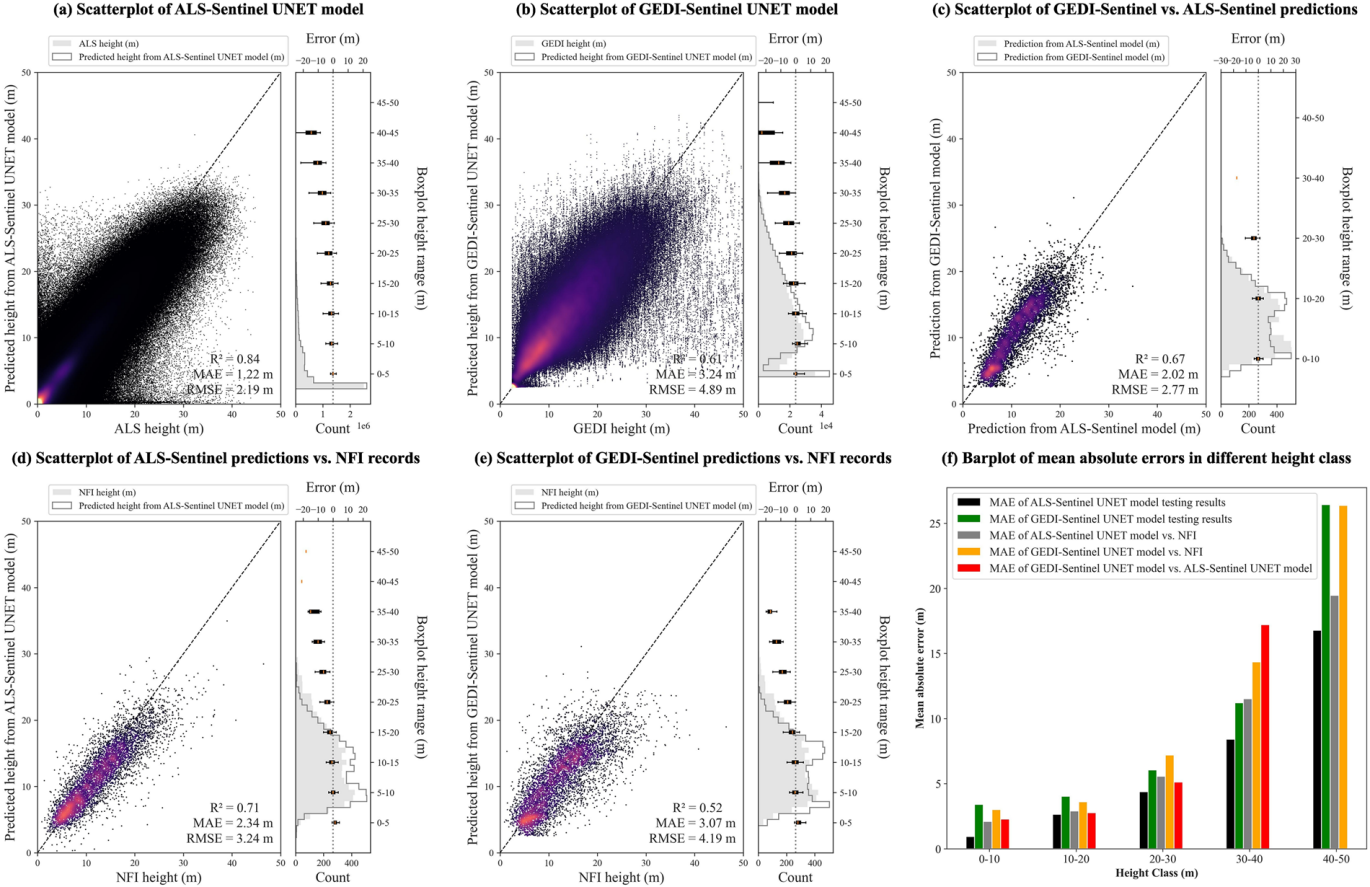
Performance of UNET canopy height models. Plot a and b are the testing scatterplot of ALS-Sentinel based and GEDI-Sentinel based UNET canopy height models, respectively. The X-axes in plot a and b are the observed canopy height in ALS and GEDI data, respectively, and the Y-axes in plot a and b are the prediction from the models. Plot c is the scatterplot that compares the ALS-Sentinel based and GEDI-Sentinel based UNET model at the location of NFI records, the X-axis is the prediction from ALS-Sentinel based UNET model, while the Y-axis is the prediction from GEDI-Sentinel based UNET model. Plot d and e compare the ALS-Sentinel based and GEDI-Sentinel based UNET model predictions and the NFI observations, respectively. The X-axes in plot d and e are the observed canopy height from NFI, while the Y-axes in plot d and e are the predicted canopy height by ALS-Sentinel based and GEDI-Sentinel based UNET model, respectively. The boxplots on the right side of those plots indicate the mean absolute errors in each height class. Plot f shows the MAE of different height classes for different comparisons.

In the methodology section, we detailed how 20% of the data was reserved as an out-of-box dataset for final evaluation of the UNET models applied to canopy height mapping. The ALS-Sentinel based UNET model demonstrated a strong performance with an R^2^ value of 0.84 and an MAE of 1.26 m (Fig. 5a), whereas the GEDI-Sentinel based UNET model achieved a lower R^2^ of 0.61 and a higher MAE of 3.24 m (Fig. 5b). When assessing the performance across various vegetation height categories (Fig. 5f), the ALS-Sentinel based model exhibited an MAE of 0.94 m for vegetation ranging from 0–10 m in height, 2.65 m for 10–20 m, 4.38 m for 20–30 m, and 8.41 m for 30–40 m. On the other hand, the GEDI-Sentinel based model recorded an MAE of 3.40 m for vegetation 0–10 m tall, 4.03 m for 10–20 m, 6.05 m for 20–30 m, and 11.21 m for 30–40 m. This analysis revealed that the ALS-Sentinel based model outperforms the GEDI-Sentinel based model, particularly in accurately mapping lower and higher vegetation heights.

For external validation, we aligned the canopy height estimates from both ALS-Sentinel based and GEDI-Sentinel based UNET models with NFI observations from the years 2017 to 2019. Given that the NFI dataset’s plots are 50 m in diameter, we adapted the predicted canopy height maps for these years to a 50 m resolution, as illustrated in Fig. 1. This adaptation involved upscaling the original 10 m resolution maps by selecting the maximum value within each 50 m pixel to represent its canopy height. Our analysis revealed that the ALS-Sentinel based model achieved an R^2^ of 0.71 and an MAE of 2.34 m when compared to NFI observations from 2017 to 2019 (Fig. 5d). In contrast, the GEDI-Sentinel based UNET model recorded an R^2^ of 0.52 and an MAE of 3.07 m, as depicted in (Fig. 5e). The bias of the ALS-Sentinel based and GEDI-Sentinel based UNET model were −1.04 meter and −1.26 m, respectively (Supplementary Figure 6c,d). Despite the ALS-Sentinel based model demonstrating superior performance over the GEDI-Sentinel based model, both models exhibited satisfactory capabilities in estimating canopy heights. In GEDI dataset, as we excluded footprints on slopes greater than 10 degrees to minimize the risk of significant errors in the measurements, to assess the validity of the GEDI-Sentinel based UNET model in areas beyond this slope, we calculated the prediction error, which we defined as the difference between the heights predicted by the model and those observed in NFI, across varying slope categories. Our results indicate that the model maintains robustness up to a 35-degree slope (Supplementary Figure 7). Beyond this slope, the vegetation types may differ significantly from those in the model’s training dataset, potentially affecting accuracy. It’s worth noting that vegetated regions with slopes exceeding 35 degrees are relatively uncommon. Additionally, we found that the ALS-Sentinel based model tends to be more reliable in these steeper regions.

We also evaluated our results by comparing them with global canopy height maps from existing literature^5,20^, both upscaled to 50 m resolution, in conjunction with observations from the 4^th^ census of the Spanish NFI. We assessed Potapov’s 2019 map^5^ against NFI data from the same year. Although Lang’s map^20^ is from 2020, we compared it to the 2019 NFI data due to the absence of 2020 NFI data. As depicted in Supplementary Figure 6, we assessed the performance of these externally sourced products against our own. Lang’s map achieved an R^2^ of 0.34, an MAE of 3.81 m, and a bias of 1.61 m, whereas Potapov’s map resulted in an R^2^ of 0.04, an MAE of 4.40 m, and a bias of −3.24 m. Our analysis demonstrates that our ALS-Sentinel based and GEDI-Sentinel based UNET canopy height models outperformed these external benchmarks in canopy height mapping accuracy.

In addition to those statistical metrics, we conducted a visual comparison between our maps and those created by Potapov and Lang. We randomly selected four small regions, as depicted in Supplementary Figures 8–11, to assess the accuracy and detail of our regional models (ALS/GEDI UNET models) against the global models from Potapov and Lang. The results suggested that the predictions from our regional models surpassed those of the global products in terms of sharpness. Furthermore, unlike the Potapov and Lang models, our UNET models are capable of directly identifying different land uses without the need for supplementary land cover masks. This capability not only simplifies the mapping process but also enhances the accuracy and robustness of the canopy height assessments across diverse landscapes. This direct approach reduces potential errors and complexities associated with the application of external land cover classifications, thereby offering a more efficient and reliable method for ecological and geographical studies.

Regarding the accuracy of our UNET tree coverage ratio model, the final testing using 20% out-of-box data yielded an R^2^ of 0.42 and an MAE of 0.16 (Supplementary Figure 5d).

**Fig. 6:**
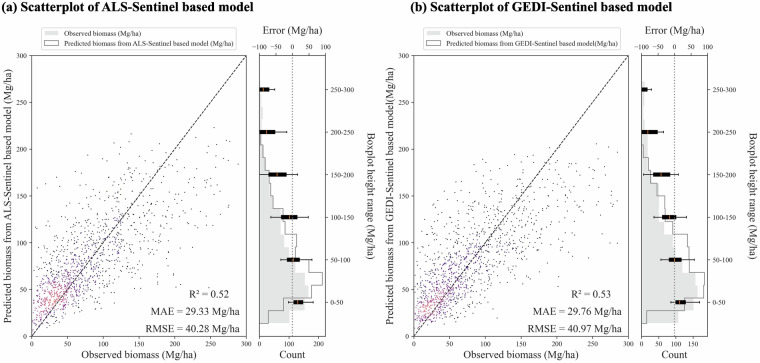
Performance of AGB RF models. Plot a and b are the testing scatter plot of ALS-Sentinel based and GEDI-Sentinel based RF AGB model, respectively. The boxplots on the right side indicate the mean absolute errors in each AGB class. The X-axes in plot a and b are the recorded ABG in NFI dataset, respectively. And the Y-axes in plot a and b are the predicted AGB from the models.

In the case of the ALS/GEDI-Sentinel based RF AGB models, the final testing results were promising. Both RF models demonstrated strong performance, achieving R^2^ values of 0.53 and recording MAE values of 29.33 Mg/ha and 29.76 Mg/ha, respectively, as shown in Fig. 6. These results surpassed those reported by Schwartz^18^, who utilized a similar UNET model for canopy height estimation but derived AGB in France using traditional allometry functions instead of machine learning techniques.

For external validation, we compared our AGB product against the global CCI-ESA map v5.0 by Santoro^64^ and the European map produced by Liu^7^ with a UNET model trained by ALS data in Europe including some campaigns in Spain. We extracted AGB data from both our maps and theirs at locations corresponding to 2019 NFI records. The comparative analysis (Supplementary Figure 12) demonstrated that our models outperformed the other maps. The differing effectiveness of the models might be due to variations in dominant forest types or tree species in the training datasets, which could influence the accuracy of AGB predictions, particularly in regions with unique forest characteristics that may not be well-represented in the global or European training data. Additionally, the visualization of our AGB maps (Supplementary Figure 13) confirmed a good correspondence with satellite imagery, further validating the efficacy of our RF AGB models. It is important to note that our AGB model may not be ideal for predicting AGB values exceeding 250 Mg/ha, as our training dataset contains few observations with such high AGB levels (Fig. 6, Supplementary Figure 12c,d).

## Usage Note

The canopy height and biomass maps provided in our dataset offer valuable insights into the canopy structure and aboveground biomass throughout the Iberian Peninsula. They also enable monitoring of forest disturbances caused by logging, fires, diseases, and the resulting impacts on height and biomass (Figs. 3c,f, 4c,f). Illustrated in Supplementary Figures 14, 15, these maps highlight areas where disturbances in forest and biomass are detected by previous studies^65^. Please note that this canopy height and biomass product is not restricted to forested areas. Users aiming to conduct forest-specific analyses are encouraged to apply a forest mask appropriate to their study objectives.

It should be note that, we derived AGB on a 50 m resolution grid, aligning with the NFI plot diameter, which is also 50 m. While this practical choice simplifies implementation, it slightly overestimates the actual NFI plot area (~45 m equivalent for circular plots) or underestimates the AGB. Users estimating total biomass across a region may consider applying an appropriate scaling factor to account for this difference.

Overall, both the ALS-Sentinel based and GEDI-Sentinel based canopy height models and AGB models demonstrated satisfactory accuracy. While the ALS-Sentinel based models slightly outperformed the GEDI-Sentinel based models in terms of metrics, they are limited by smaller spatial coverage and higher costs associated with data collection. In contrast, the GEDI model offers extensive spatial coverage and benefits from continuous annual updates. This large coverage allows for the application of consistent methodologies to monitor forest disturbances over time using GEDI data.

## Supplementary information

Supplementary documentary

## Data Availability

The code that used in this study can be found in Figshare^66^.
