# Supplementary material for: Canopy height and biomass distribution across the forests of Iberian Peninsula
Source: Sci Data. 2025 Apr 22;12:678. doi: 10.1038/s41597-025-05021-9 (PMC12015371; doi:10.1038/s41597-025-05021-9)
Supplement: Supplementary file 1 — Supplementary documentary [file 41597_2025_5021_MOESM1_ESM.pdf]

Supplementary document - Canopy height and biomass distribution across the forests of Iberian Peninsula

Authors

Yang Su [a, b, c], Martin Schwartz [b], Ibrahim Fayad [b], Mariano García [d], Miguel A. Zavala [e], Julián Tijerín-Triviño [e], Julen Astigarraga [e], Verónica Cruz-Alonso [e, f], Siyu Liu [g], Xianglin Zhang [c, h], Songchao Chen [h,i], François Ritter [b], Nikola Besic [j], Alexandre d'Aspremont [a], Philippe Ciais [b]

Affiliations

[a] Département d'Informatique, École Normale Supérieure – PSL, 45 Rue d'Ulm, 75005 Paris, France

[b] Laboratoire des Sciences du Climat et de l'Environnement, CEA CNRS UVSQ Orme des Merisiers, 91190 Gif-sur-Yvette, France

[c] UMR ECOSYS, INRAE AgroParisTech, Université Paris-Saclay, 91120 Palaiseau, France

[d] Universidad de Alcalá, Departmento de Geología, Geografía y Medio Ambiente, Environmental Remote Sensing Research Group, 28801 Alcalá de Henares, Spain

[e] Universidad de Alcalá, Department of Life Sciences, Forest Ecology and Restoration Group (FORECO), 28805, Alcalá de Henares, Spain

[f] Department of Biodiversity, Ecology and Evolution, Complutense University of Madrid, 28040 Madrid, Spain

[g] Department of Geosciences and Natural Resource Management, Copenhagen University, 1958 Frederiksberg, Denmark

[h] College of Environmental and Resource Sciences, Zhejiang University, 310058 Hangzhou, China

[i] ZJU-Hangzhou Global Scientific and Technological Innovation Center, Zhejiang University, 311215 Hangzhou, China

[j] IGN, ENSG, Laboratoire d'inventaire forestier (LIF), 54000 Nancy, France

Corresponding Author

Yang Su yang.su@ens.fr +33 1 89 10 07 67 École Normale Supérieure – PSL

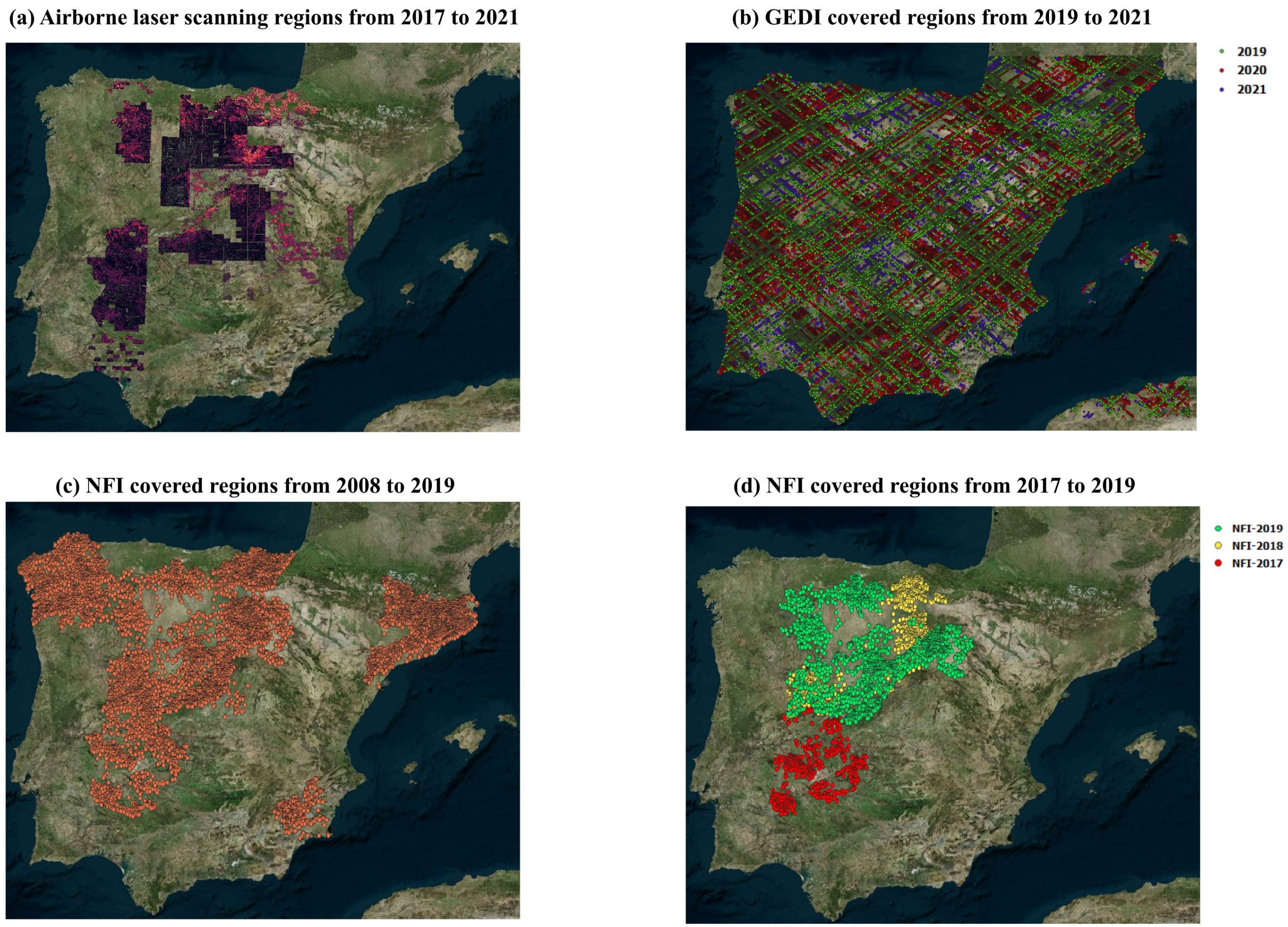

Supplementary figure 1 | Spatial coverage of ALS and GEDI datasets. Plot a is the coverage of ALS data [1] from 2017 to 2021 (205213.2 km^2), plot b is the coverage of GEDI data [2] from 2019 to 2021 (in total 2,579,421 footprints), plot c is the coverage of NFI data [3] from 2008 to 2019 (in total 16566 plots), and plot d is the coverage of NFI data from 2017 to 2019 (in total 6308 plots).

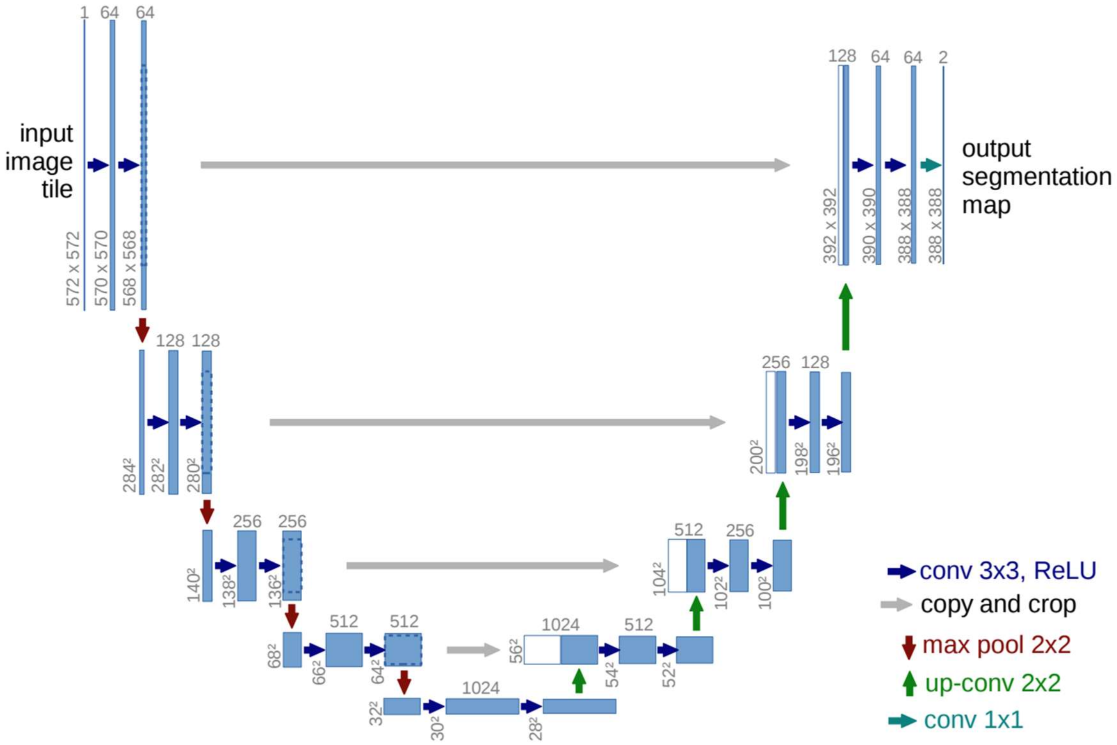

Supplementary figure 2 | Structure of UNET model [4]. The U-Net architecture, a convolutional neural network primarily used for image segmentation, consists of a contracting path, bottleneck, and expansive path. Initially, the input image is processed through consecutive 3x3 convolutional layers with ReLU activations, followed by 2x2 max pooling to reduce spatial dimensions while doubling feature channels from 64 to 1024. The bottleneck, featuring further convolutions, bridges to the expansive path where 2x2 up-convolutions increase spatial dimensions and feature concatenation from corresponding contracting layers enhances localization accuracy. The network concludes with a 1x1 convolution that outputs the segmentation map with the desired number of classes, typically for binary segmentation tasks. This efficient structure allows U-Net to excel in medical image segmentation by effectively integrating contextual and spatial information, even with limited data.

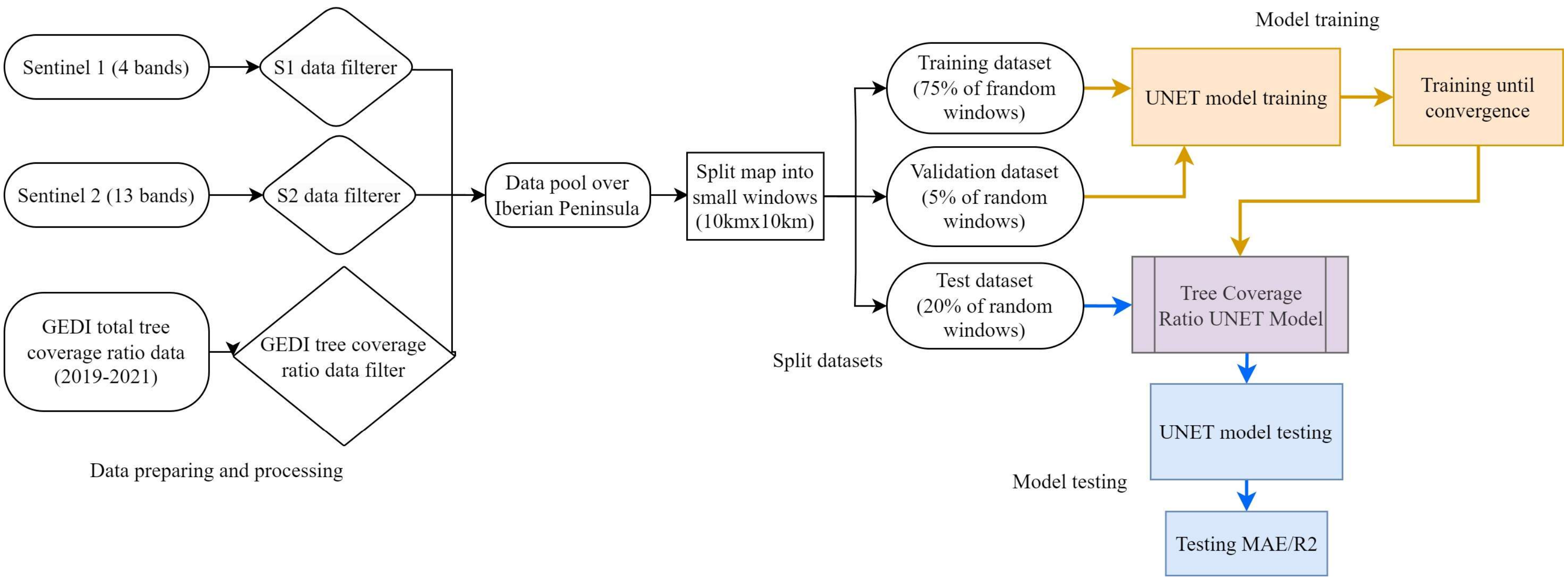

Supplementary figure 3 | Flow chart of training, testing and validation of UNET tree coverage ratio model. This plot showed the algorithm of model training (marked by orange color) and testing (marked by blue color) for UNET tree coverage ratio model, which is used to generate the necessary data for AGB model training.

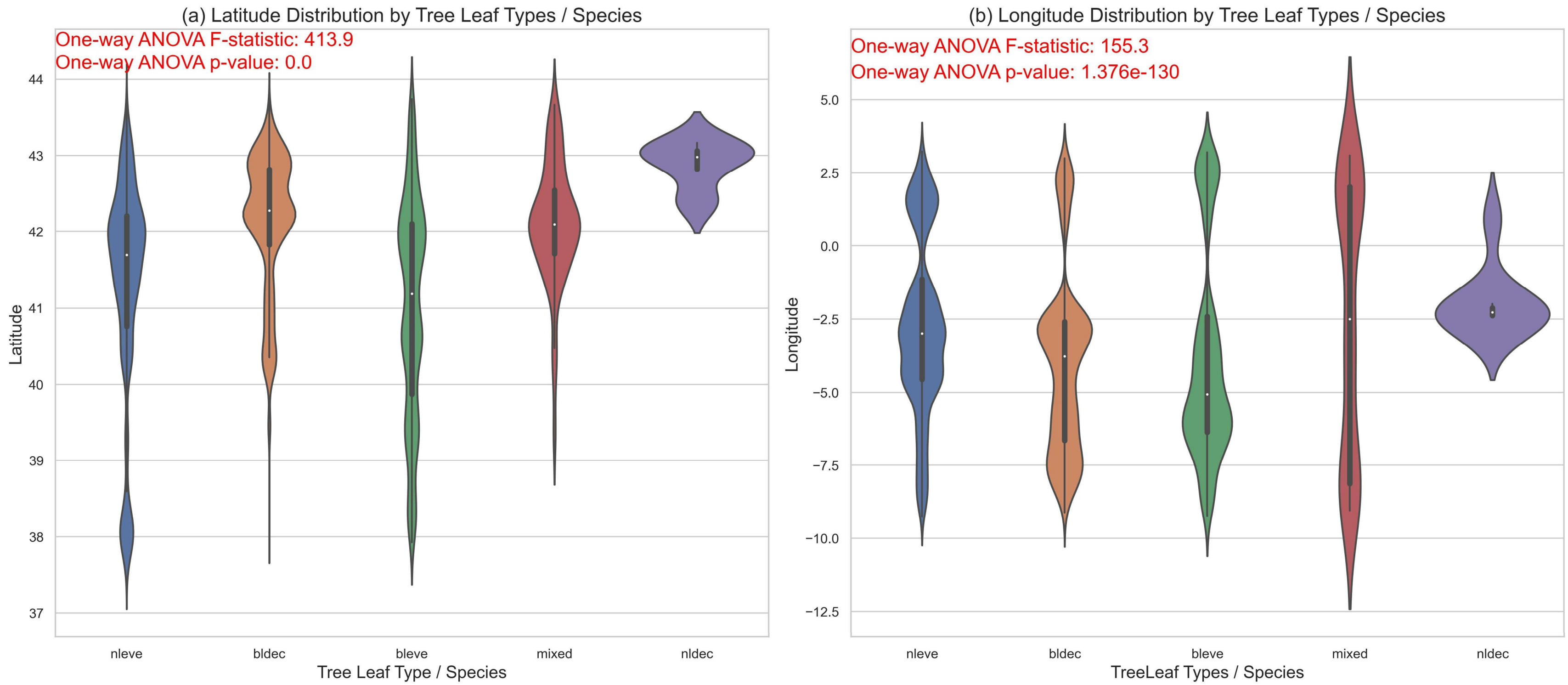

Supplementary figure 4 | One-way ANOVA test and violin plot. This plot showed the one-way ANOVA test of the species with different tree leaf types and latitude/longitude., which indicate whether there are significant differences in the average latitude and longitude among different tree species. In our case, the small p-value (close to zero) strongly suggests that the differences in mean latitude among different tree species are statistically significant. And a higher F-statistic indicates a greater variance among the group means relative to the variance within the groups, indicating a more pronounced difference in the mean latitudes among different tree species compared to longitude. ‘nleve’ is the needle leaf evergreen; ‘bldec’ is the broad leaf deciduous; ‘bleve’ is the broad leaf evergreen; ‘nldec’ is the needle leaf deciduous; ‘mixed’ is the mixed leaf types.

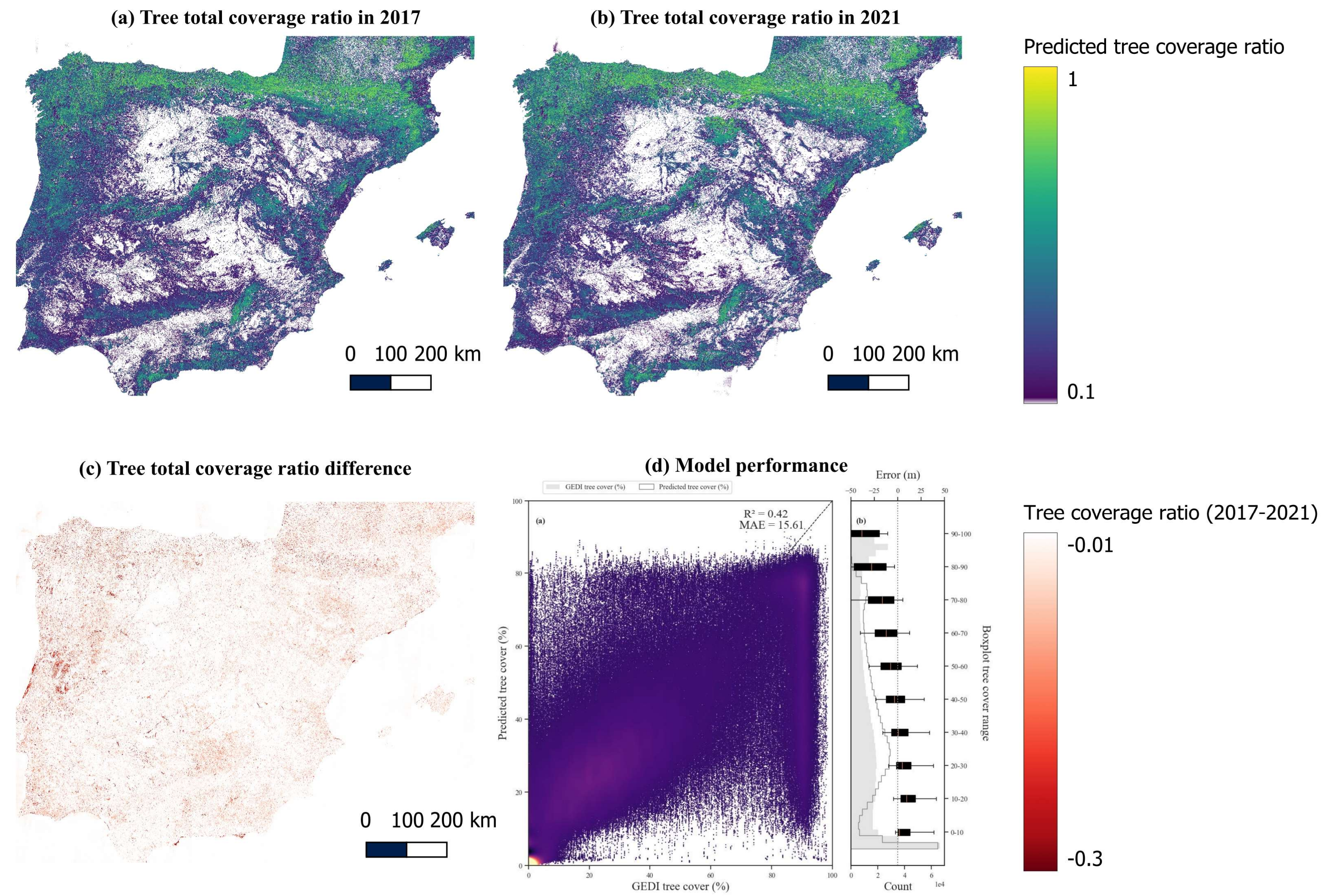

Supplementary figure 5 | Tree coverage ratio maps and the model performance. Plot a and b are the tree coverage ratio in 2017 and 2021 predicted by UNET tree coverage ratio model, respectively. Plot c is the tree coverage ratio difference predicted by the UNET tree coverage ratio model. Plot d is the model performance of the UNET tree coverage ratio model.

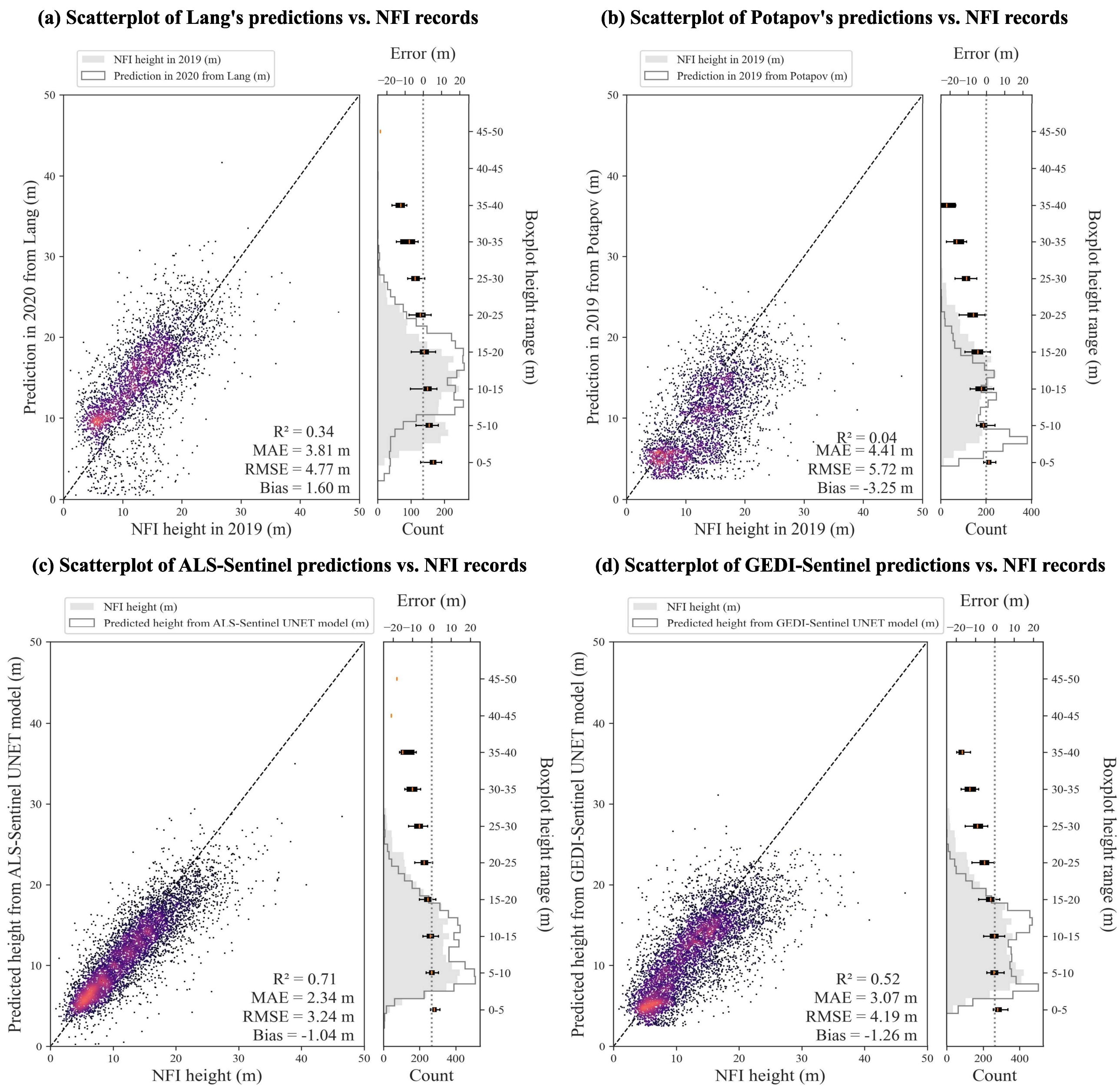

Supplementary figure 6 | The comparison of ALS and GEDI predictions of canopy height with the products from Lang [5] and Potapov [6]. Plot a is the scatter plot of predicted canopy heights of Lang in 2020 versus the NFI recorded canopy heights of 2019. Plot b is the scatter plot of predicted canopy heights of Potapov in 2019 versus the NFI recorded canopy heights of 2019. Plot c is the scatter plot of predicted canopy heights of our ALS-based UNET canopy height model in 2019 versus the NFI recorded canopy heights of 2019. Plot d is the scatter plot of predicted canopy heights of our GEDI-based UNET canopy height model in 2019 versus the NFI recorded canopy heights of 2019. The boxplots on the right side indicate the mean absolute errors in each height class.

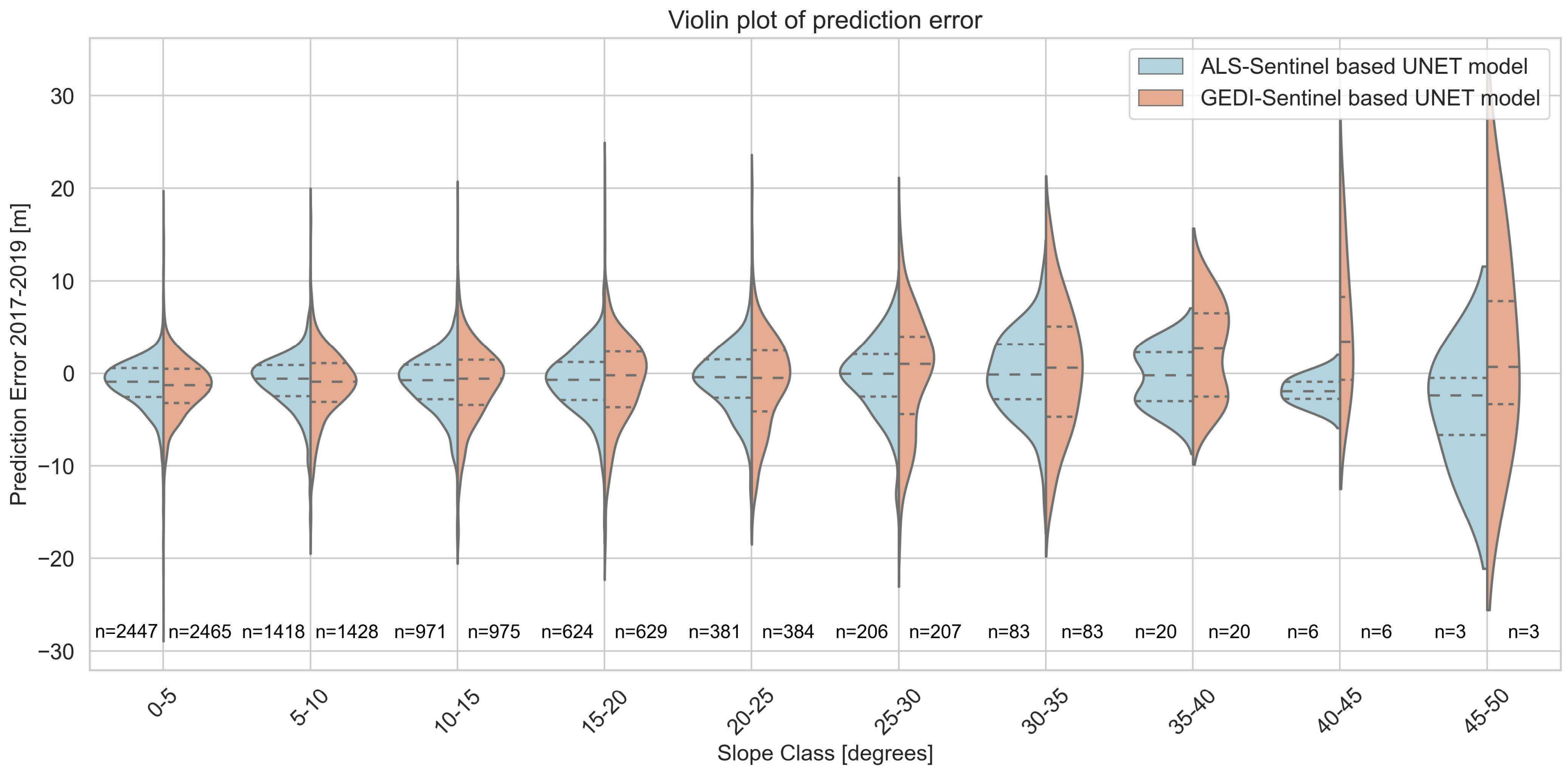

Supplementary figure 7 | Violin plot of prediction error from ALS and GEDI based UNET model. This plot is the violin plot of prediction error of different slope classes [7], where the prediction error is defined as the prediction from ALS (in light blue), or GEDI (in orange) based UNET model minus the NFI observation. The dash lines inside the violin plots are the 75th, 50th (median), and 25th percentile line, respectively.

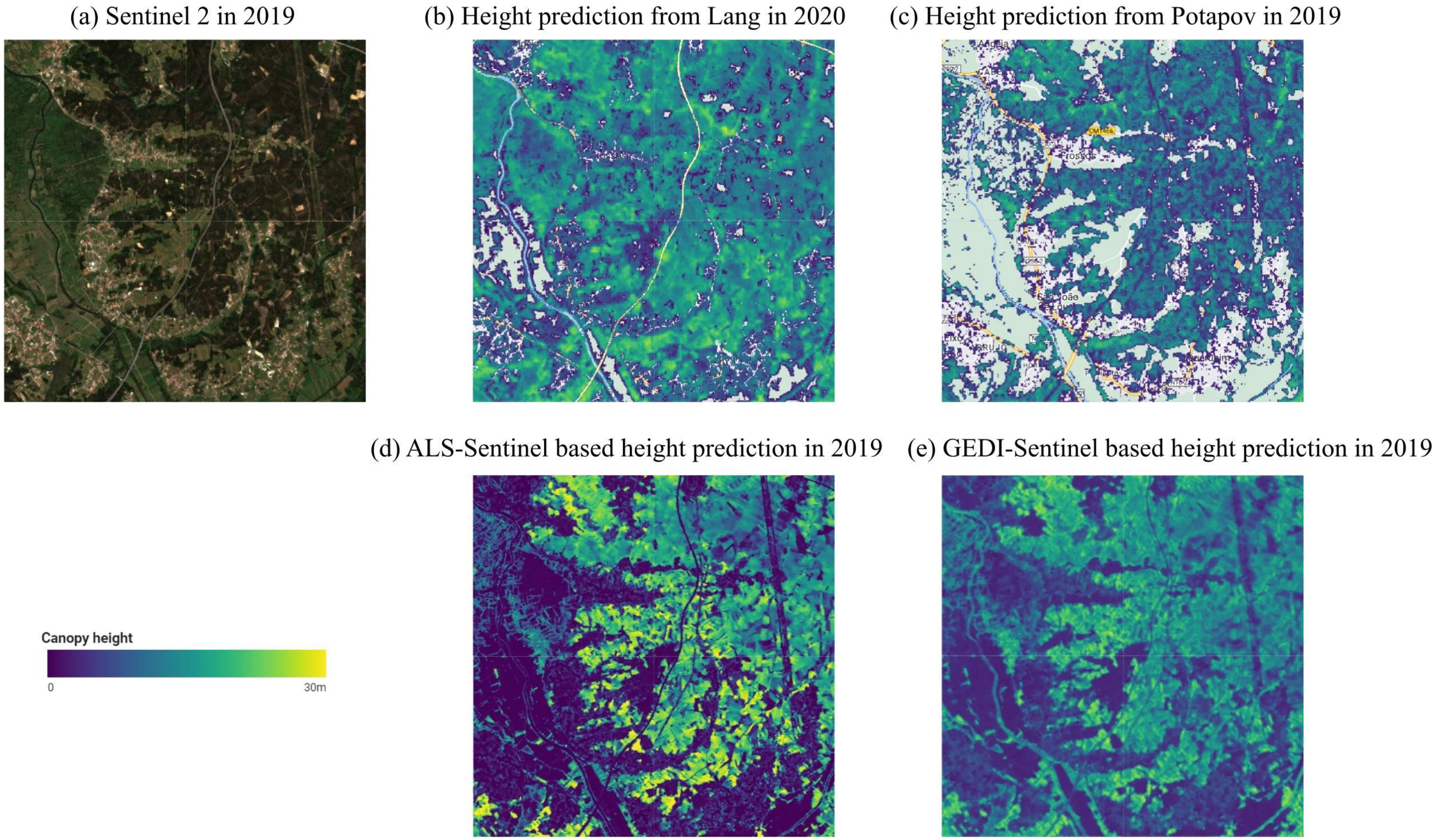

Supplementary figure 8 | Comparison of our products to Lang's products [8] and Potapov's products [6] - case 1. Plot a is the sentinel images in 2019. Plot b is the tree height prediction from Lang in 2020. Plot c is the tree height prediction from Potapov in 2019. Plot c is the tree height prediction from our ALS-based UNET canopy height model in 2019. Plot d is the tree height prediction from our GEDI-based UNET canopy height model in 2019.

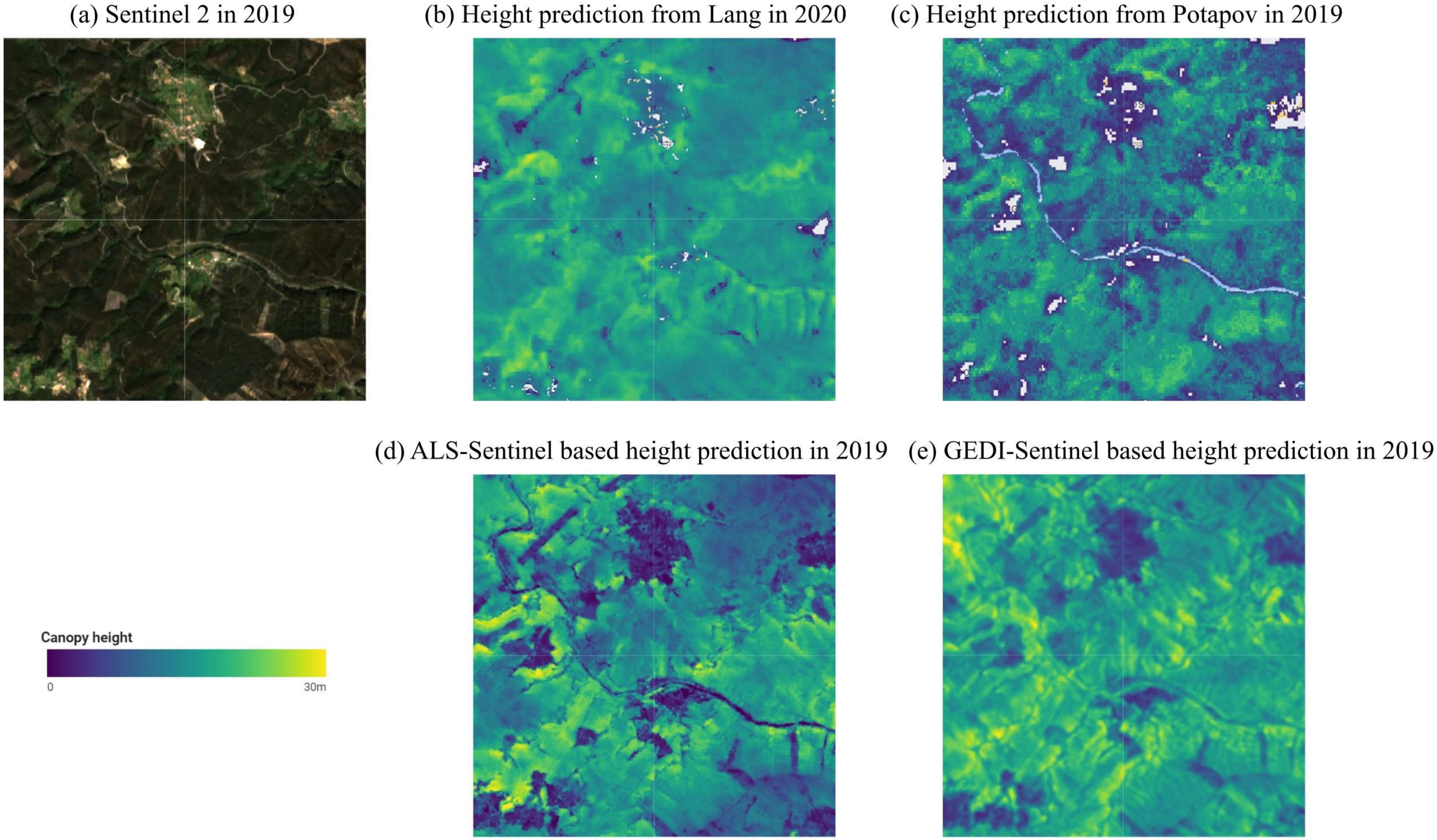

Supplementary figure 9 | Comparison of our products to Lang's products [8] and Potapov's products [6] - case 2. Plot a is the sentinel images in 2019. Plot b is the tree height prediction from Lang in 2020. Plot c is the tree height prediction from Potapov in 2019. Plot c is the tree height prediction from our ALS-based UNET canopy height model in 2019. Plot d is the tree height prediction from our GEDI-based UNET canopy height model in 2019.

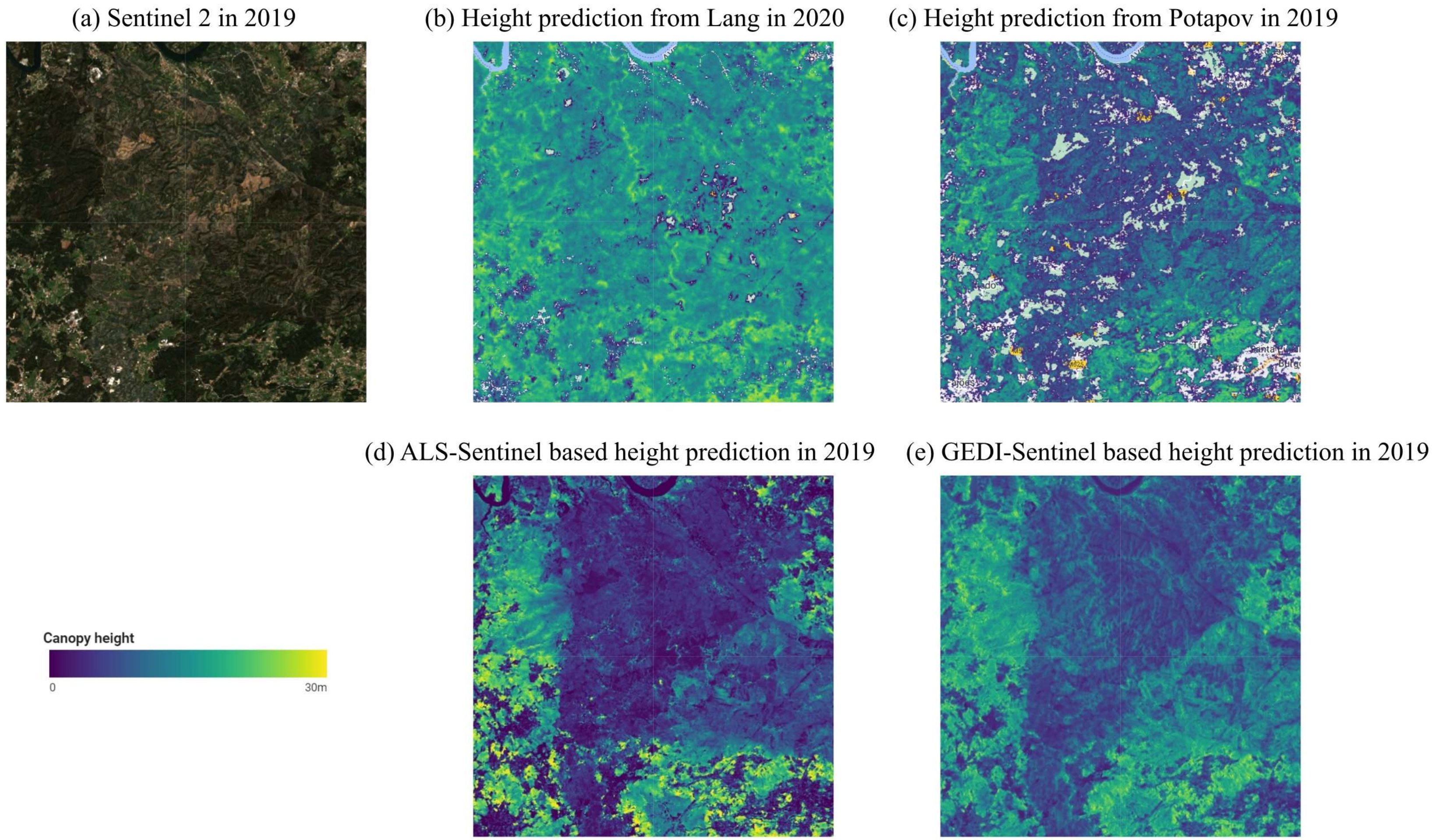

Supplementary figure 10 | Comparison of our products to Lang's products [8] and Potapov's products [6] - case 3. Plot a is the sentinel images in 2019. Plot b is the tree height prediction from Lang in 2020. Plot c is the tree height prediction from Potapov in 2019. Plot c is the tree height prediction from our ALS-based UNET canopy height model in 2019. Plot d is the tree height prediction from our GEDI-based UNET canopy height model in 2019.

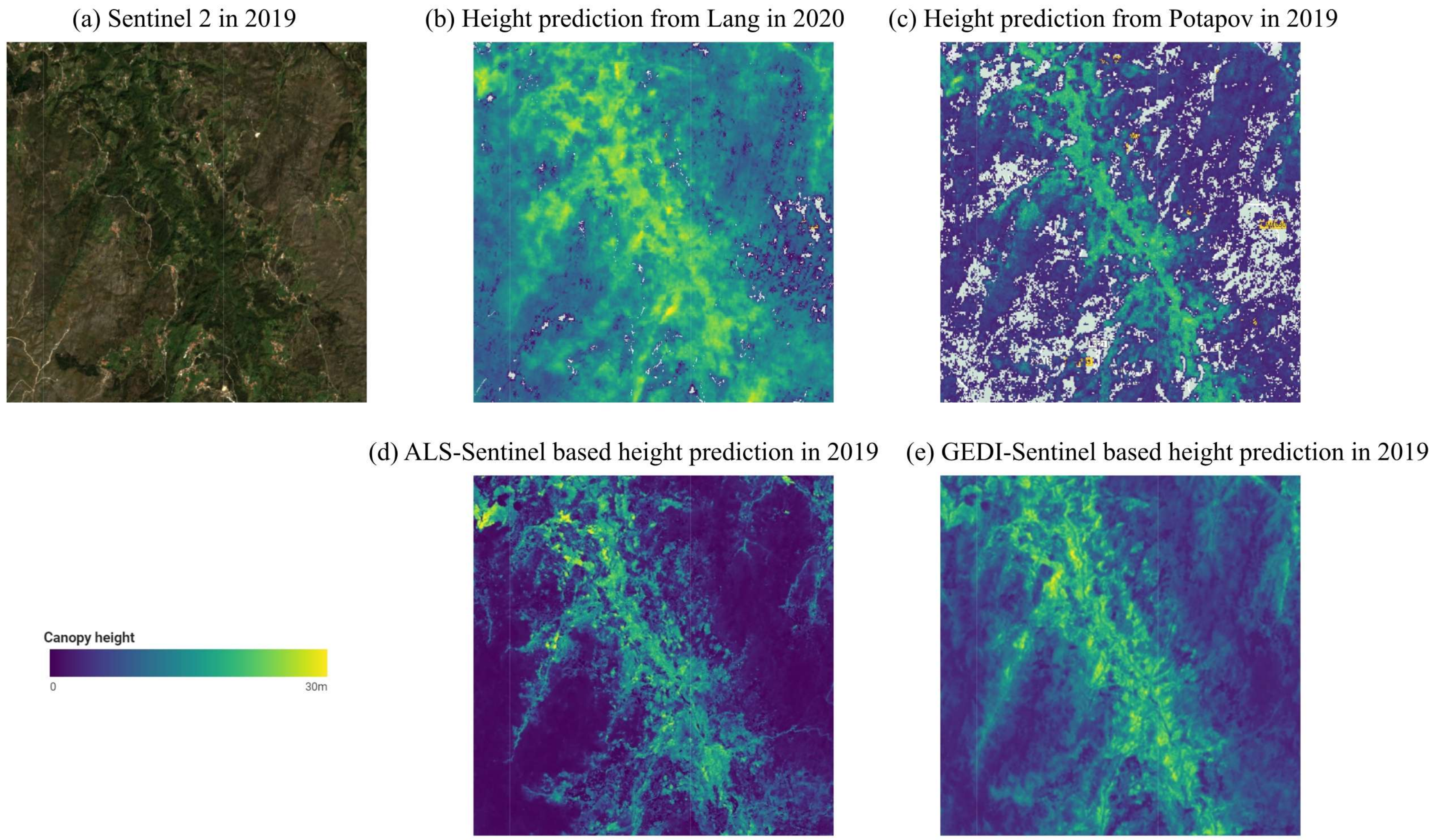

Supplementary figure 11 | Comparison of our products to Lang's products [8] and Potapov's products [6] - case 4. Plot a is the sentinel images in 2019. Plot b is the tree height prediction from Lang in 2020. Plot c is the tree height prediction from Potapov in 2019. Plot c is the tree height prediction from our ALS-based UNET canopy height model in 2019. Plot d is the tree height prediction from our GEDI-based UNET canopy height model in 2019.

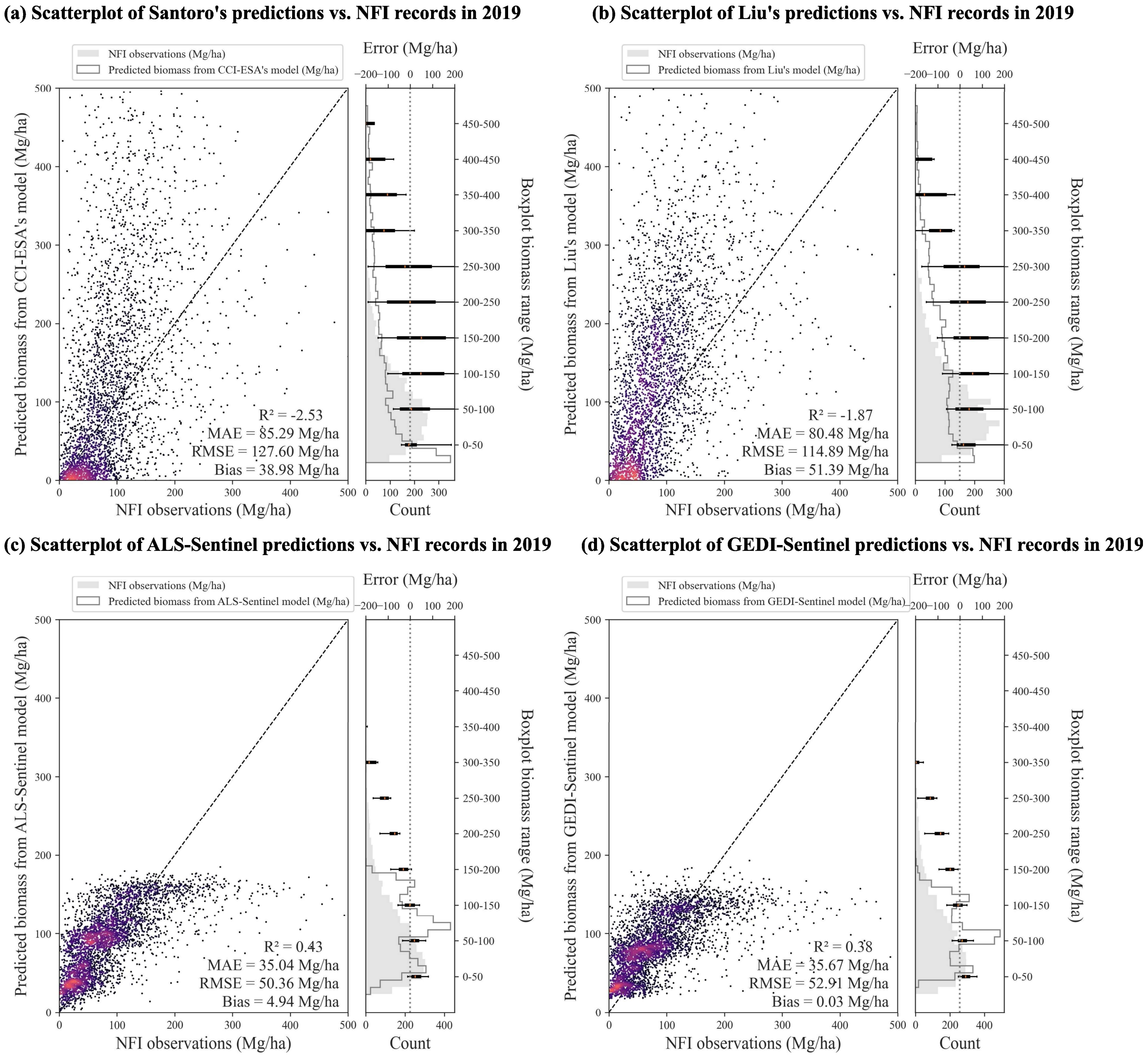

Supplementary figure 12 | The comparison of ALS and GEDI AGB predictions in 2019 with the products from Liu [9] and Santoro [10]. Plot a is the scatter plot of predicted AGB of Santoro in 2019 versus the NFI recorded AGB of 2019. Plot b is the scatter plot of predicted AGB of Liu in 2019 versus the NFI recorded AGB of 2019. Plot c is the scatter plot of predicted AGB of our ALS-based RF AGB model in 2019 versus the NFI recorded AGB of 2019. Plot d is the scatter plot of predicted AGB of our GEDI-based RF AGB model in 2019 versus the NFI recorded AGB of 2019. The boxplots on the right side indicate the mean absolute errors in each AGB class.

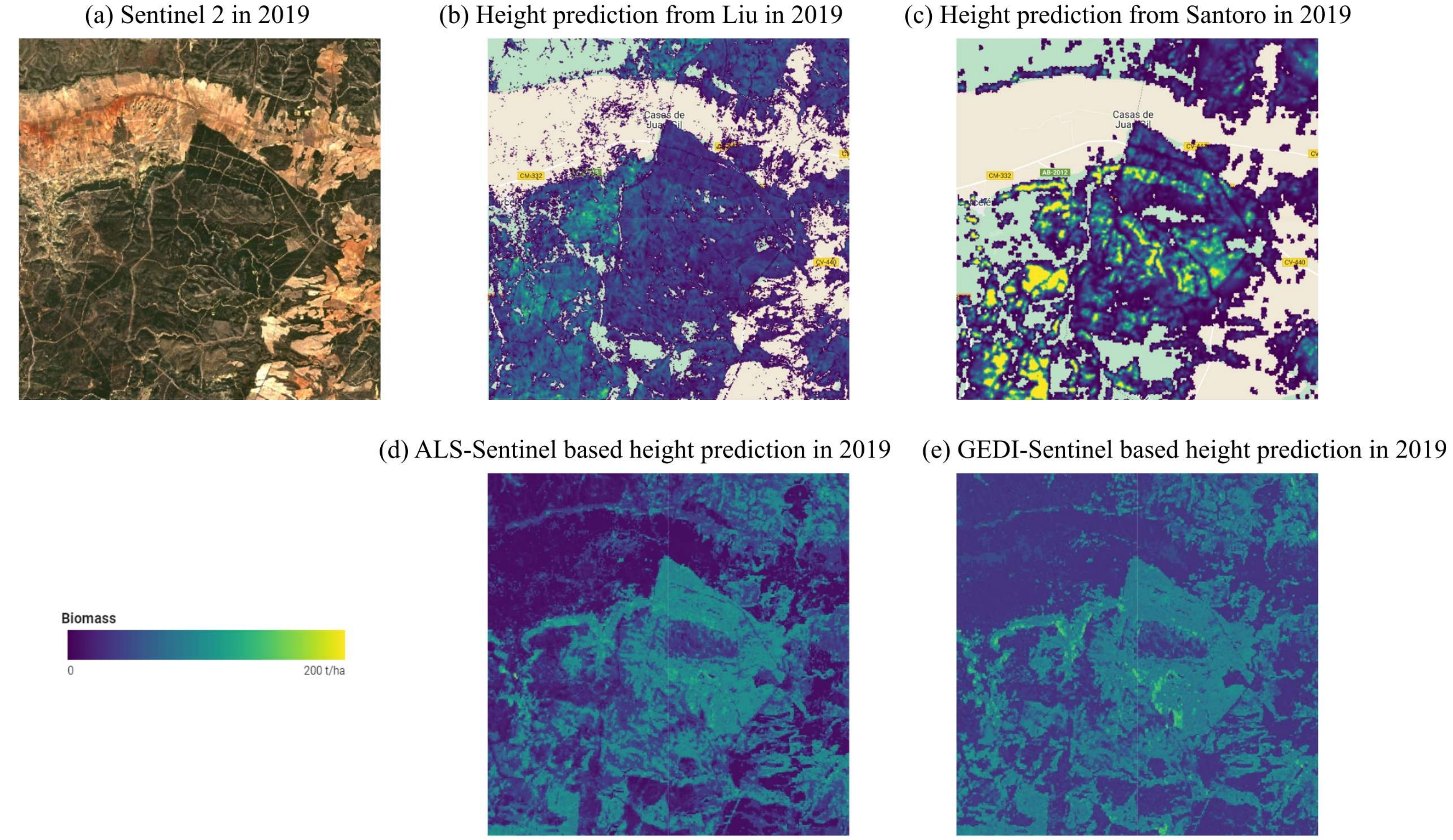

Supplementary figure 13 | The comparison of ALS and GEDI biomass predictions with the products from Liu [9] and Santoro [9]. Plot a is the sentinel images in 2019. Plot b is the AGB prediction from Liu in 2020. Plot c is the AGB prediction from Santori in 2019. Plot c is the AGB prediction from our ALS-based AGB RF model in 2019. Plot d is the AGB prediction from our GEDI-based AGB RF model in 2019.

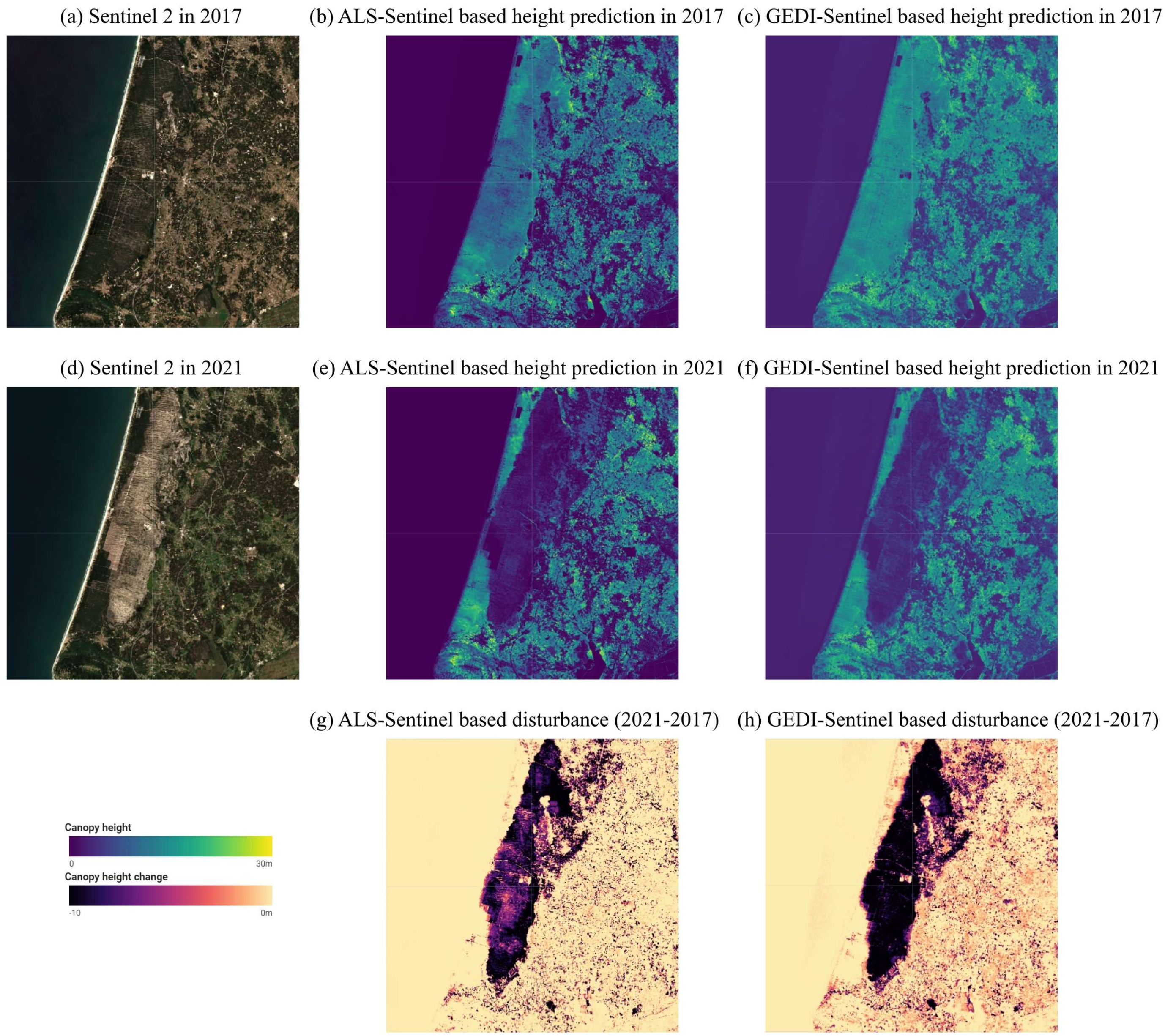

Supplementary figure 14 | Forest disturbance detection. Plot a is the S2 images in 2017. Plot b is the tree height prediction from our ALS-based UNET canopy height model in 2017. Plot c is the tree height prediction from our GEDI-based UNET canopy height model in 2017. Plot d is the S2 images in 2021. Plot e is the tree height prediction from our ALS-based UNET canopy height model in 2021. Plot f is the tree height prediction from our GEDI-based UNET canopy height model in 2021. Plot g is the disturbance detected by our ALS-based UNET canopy height model from 2017 to 2021. Plot h is the disturbance detected by our GEDI-based UNET canopy height model from 2017 to 2021.

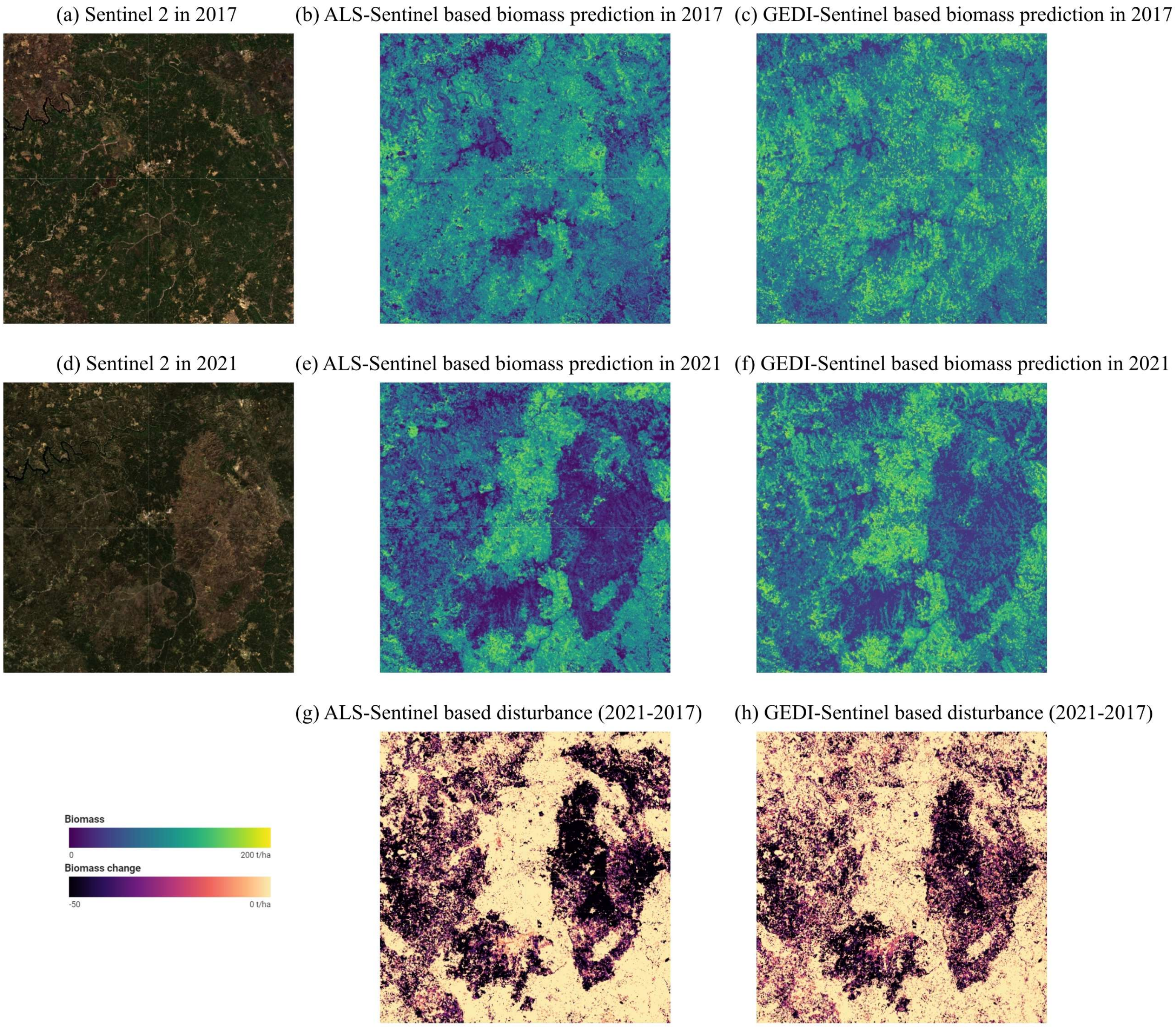

Supplementary figure 15 | Biomass disturbance detection (these disturbances were also detected by Guerra-Hernandez [11]). Plot a is the S2 images in 2017. Plot b is the AGB prediction from our ALS-based AGB RF model in 2017. Plot c is the AGB prediction from our GEDI-based AGB RF model in 2017. Plot d is the S2 images in 2021. Plot e is the AGB prediction from our ALS-based AGB RF model in 2021. Plot f is the AGB prediction from our GEDI-based AGB RF model in 2021. Plot g is the disturbance detected by our ALS-based AGB RF model from 2017 to 2021. Plot h is the disturbance detected by our GEDI-based AGB RF model from 2017 to 2021.

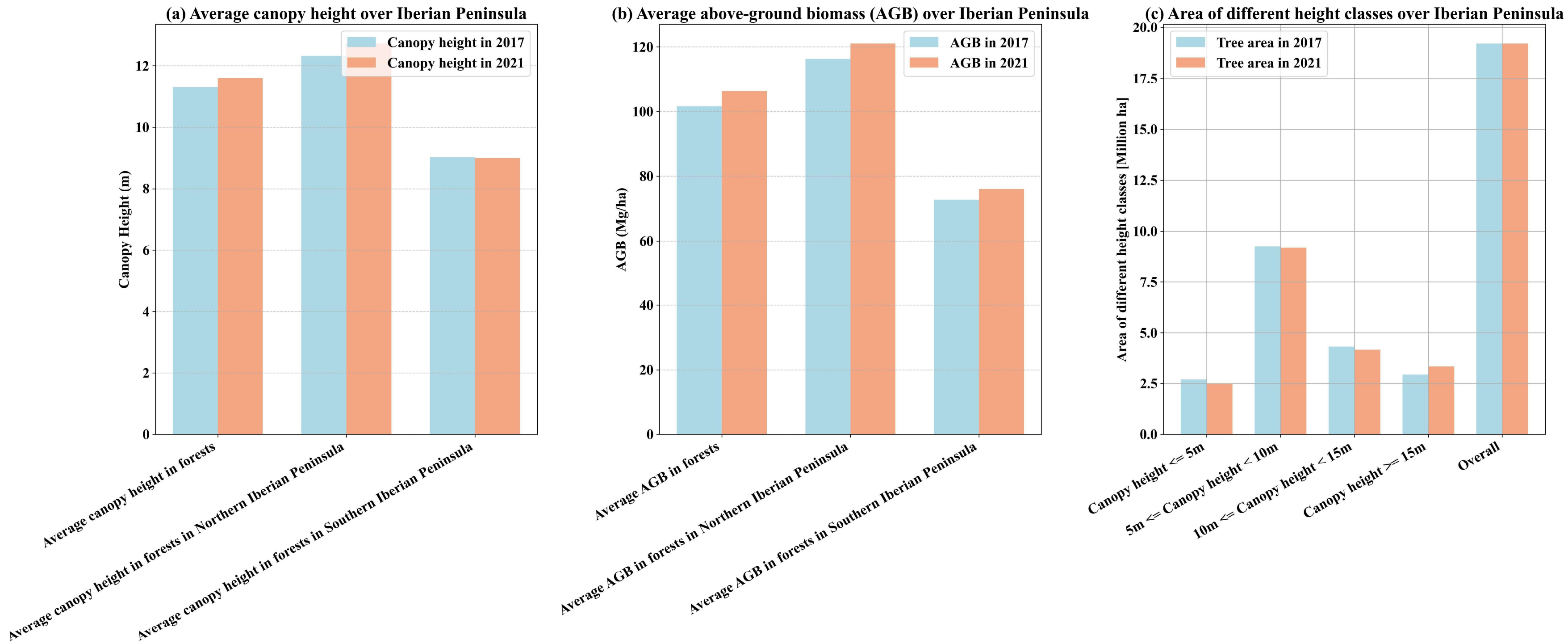

Supplementary figure 16 | Statistics of height and AGB in the forests of Iberian Peninsula in the year 2017 and 2021 (predicted by ALS-Sentinel based UNET model). Plot a is the statistics of the canopy height in the forests in the Whole/Northern/Southern Iberian Peninsula, plot b is the statistics of the AGB in the forests in the Whole/Northern/Southern Iberian Peninsula. Note that in this statistical analysis in plot a and plot b, only vegetation exceeding 5 m in height was included. Plot c is the forest area of different canopy height classes.

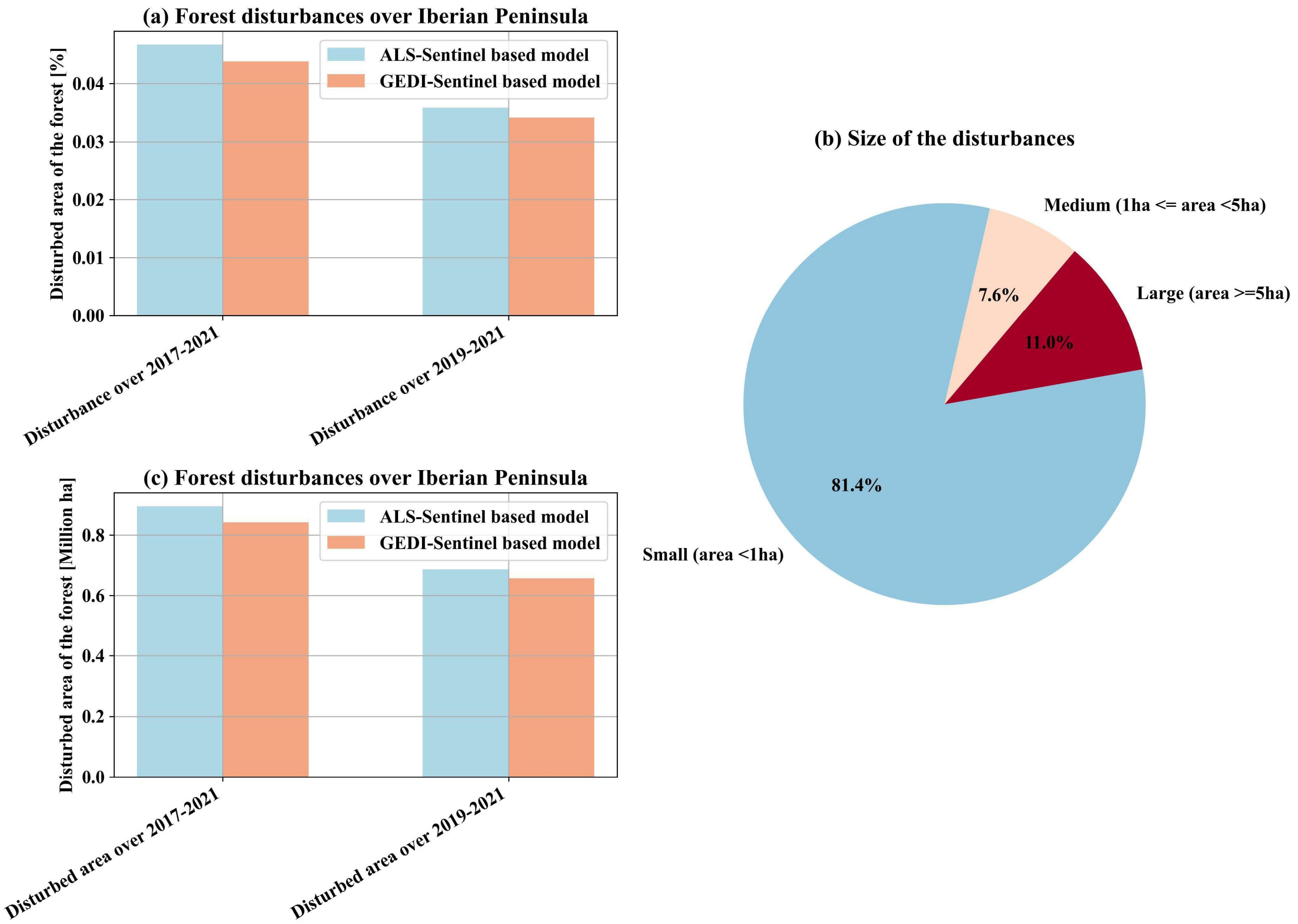

Supplementary figure 17 | Statistics of height and biomass disturbances from 2017 to 2021. Plot a is the statistics of canopy height and biomass disturbances detected by ALS-Sentinel based and GEDI-Sentinel based models over the Iberian Peninsula from 2017 to 2021. Plot b is the statistics of the size of the disturbances detected by the ALS-Sentinel based models from 2017 to 2021. Plot c is the disturbed area detected by ALS-Sentinel based and GEDI-Sentinel based models over the Iberian Peninsula from 2017 to 2021. It is important to note that a disturbance is identified only if there is a decrease in canopy height greater than 4 meters, which corresponds to the MAE of the GEDI-Sentinel based UNET canopy height model.

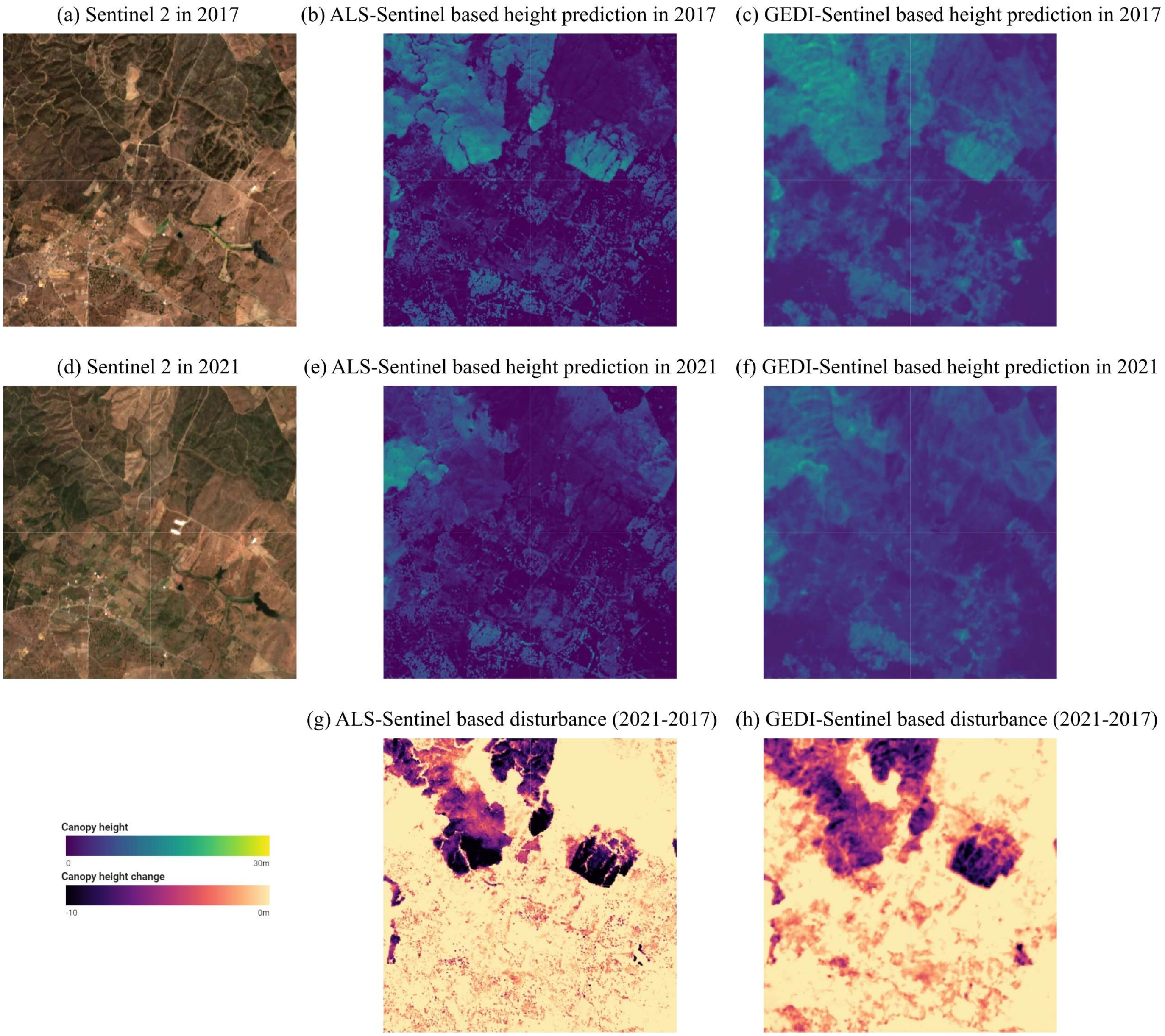

Supplementary figure 18 | Differences in GEDI and ALS. Plot a is the S2 images in 2017. Plot b is the tree height prediction from our ALS-based UNET canopy height model in 2017. Plot c is the tree height prediction from our GEDI-based UNET canopy height model in 2017. Plot d is the S2 images in 2021. Plot e is the tree height prediction from our ALS-based UNET canopy height model in 2021. Plot f is the tree height prediction from our GEDI-based UNET canopy height model in 2021. Plot g is the disturbance detected by our ALS-based UNET canopy height model from 2017 to 2021. Plot h is the disturbance detected by our GEDI-based UNET canopy height model from 2017 to 2021.

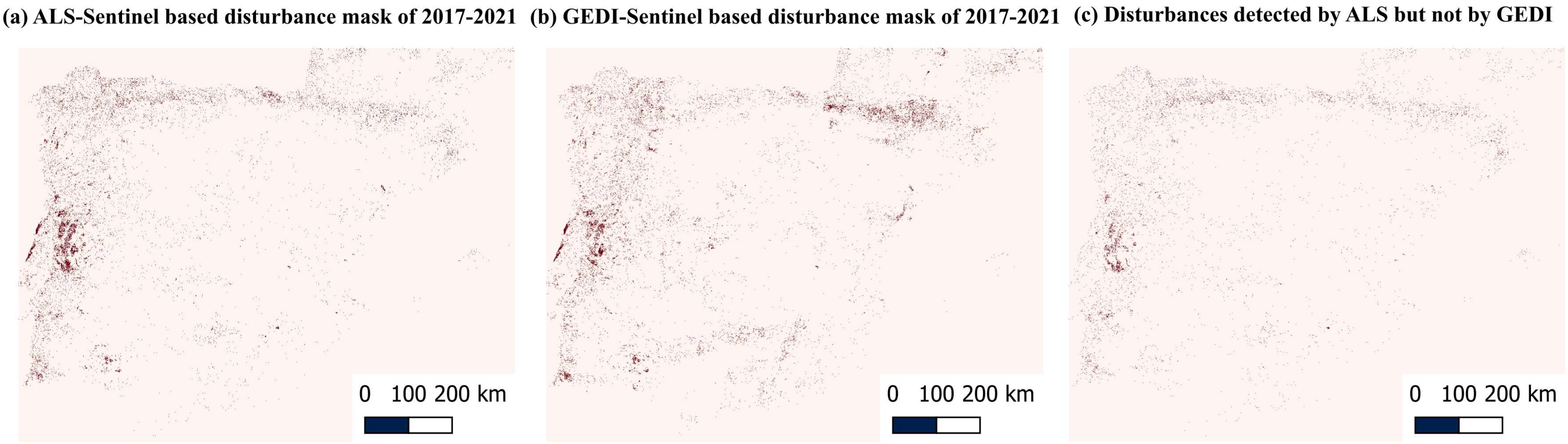

Supplementary figure 19 | Differences in GEDI and ALS disturbance mask. Plot a is disturbance mask from 2017 to 2021 produced by ALS-based UNET height model. Plot b is disturbance mask from 2017 to 2021 produced by GEDI-based UNET height model. Plot c shows the regions that were detected by ALS-based UNET model but not by GEDI-based UNET model. Note that a disturbance is identified only if there is a decrease in canopy height greater than 4 meters, which corresponds to the MAE of the GEDI-based UNET model.

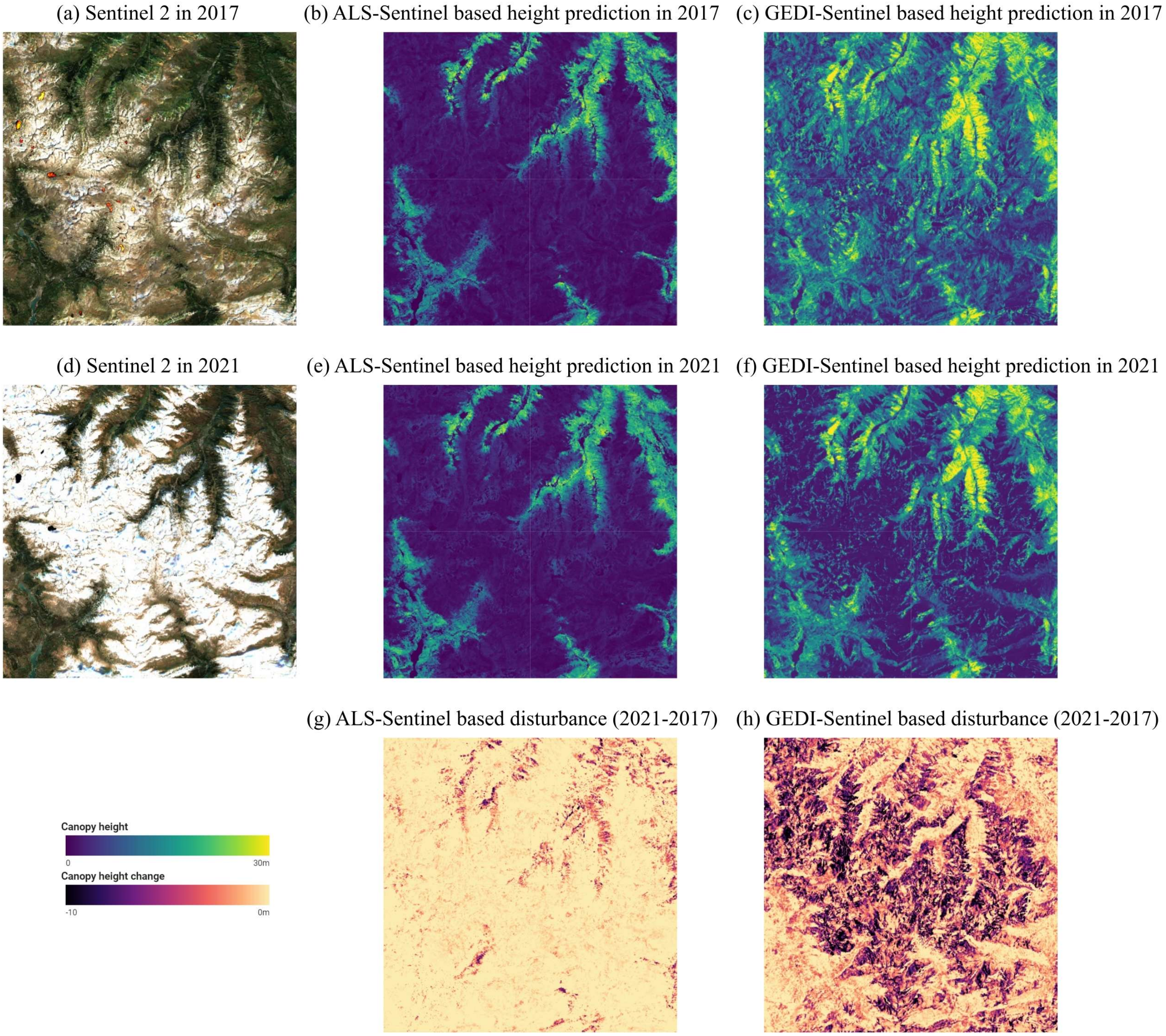

Supplementary figure 20 | Differences in GEDI and ALS in snow mountain regions. Plot a is the S2 images in 2017. Plot b is the tree height prediction from our ALS-based UNET canopy height model in 2017. Plot c is the tree height prediction from our GEDI-based UNET canopy height model in 2017. Plot d is the S2 images in 2021. Plot e is the tree height prediction from our ALS-based UNET canopy height model in 2021. Plot f is the tree height prediction from our GEDI-based UNET canopy height model in 2021. Plot g is the disturbance detected by our ALS-based UNET canopy height model from 2017 to 2021. Plot h is the disturbance detected by our GEDI-based UNET canopy height model from 2017 to 2021.

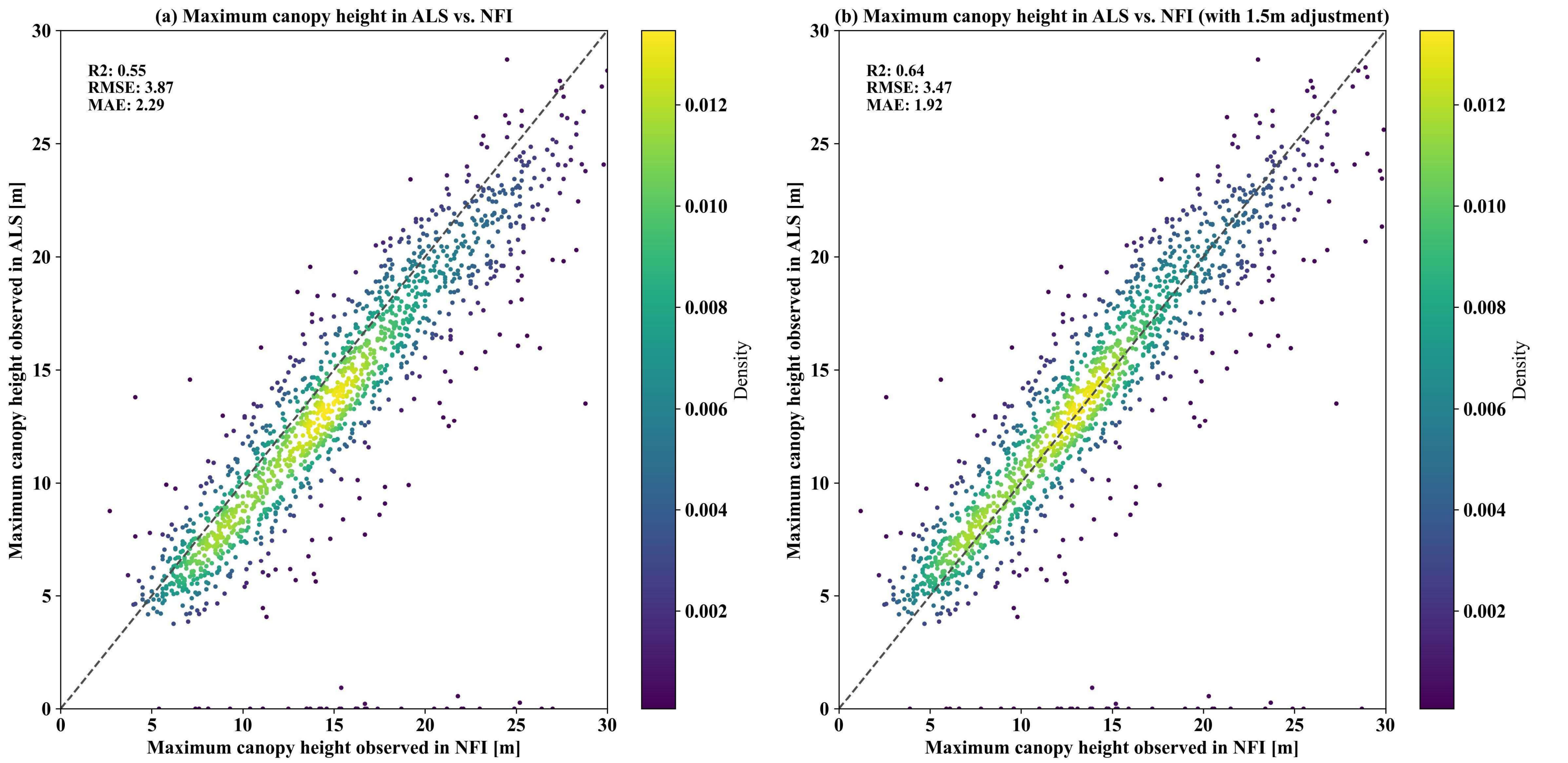

Supplementary figure 21 | Scatterplot of the maximum canopy height from observed ALS and observed NFI at the location of NFI plots. Plot a compares observed ALS and observed NFI canopy heights, where ALS maximum heights are derived from the highest value among 2 m resolution pixels within a 50 m diameter circle around each NFI plot location, corresponding to the size of the NFI plots. Plot b compares observed ALS canopy heights with corrected NFI heights, which were obtained by subtracting 1.5 m from the original observed NFI values.

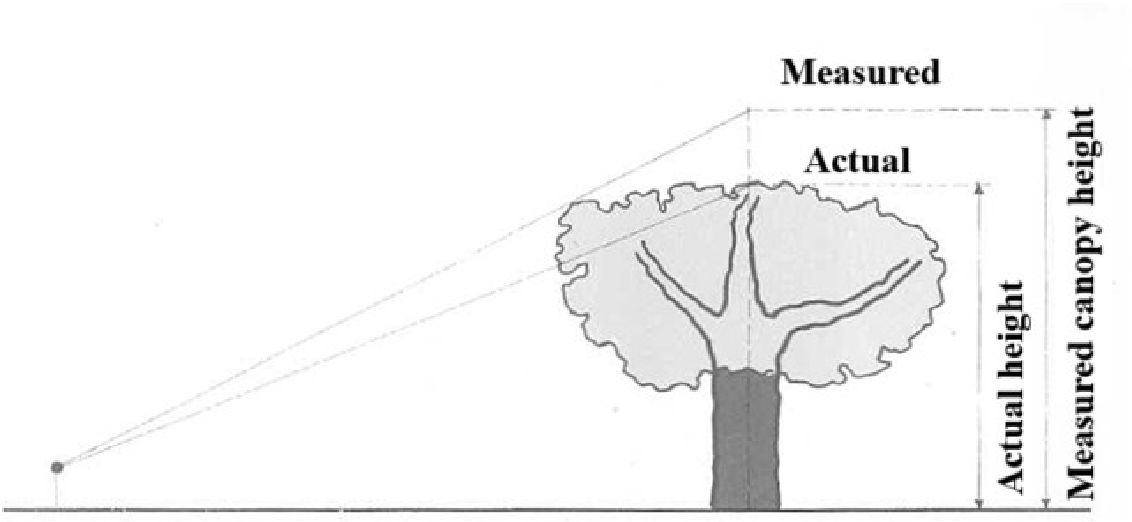

Supplementary figure 22 | Potential errors may arise in the manual measurement of canopy height (image from Villaescusa et al. [12]). This plot depicts a potential scenario in which canopy height may be overestimated.

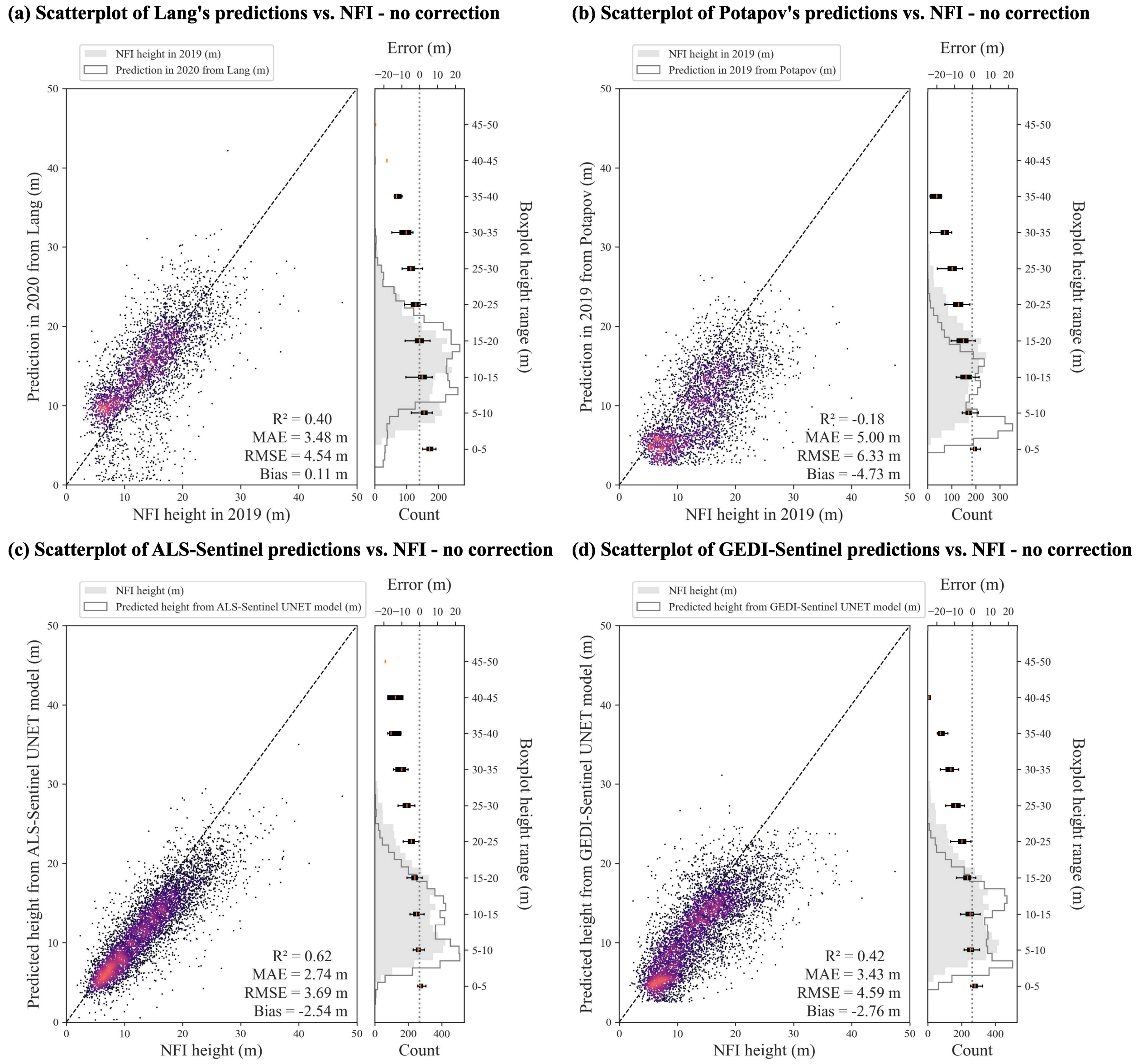

Supplementary figure 23 | The comparison of ALS and GEDI predictions of canopy height with the products from Lang [5] and Potapov [6] based on uncorrected NFI observed maximum canopy height. Plot a is the scatter plot of predicted canopy heights of Lang in 2020 versus the uncorrected NFI recorded canopy heights of 2019. Plot b is the scatter plot of predicted canopy heights of Potapov in 2019 versus the uncorrected NFI recorded canopy heights of 2019. Plot c is the scatter plot of predicted canopy heights of our ALS-based UNET canopy height model in 2019 versus the uncorrected NFI recorded canopy heights of 2019. Plot d is the scatter plot of predicted canopy heights of our GEDI-based UNET canopy height model in 2019 versus the uncorrected NFI recorded canopy heights of 2019. The boxplots on the right side indicate the mean absolute errors in each height class.

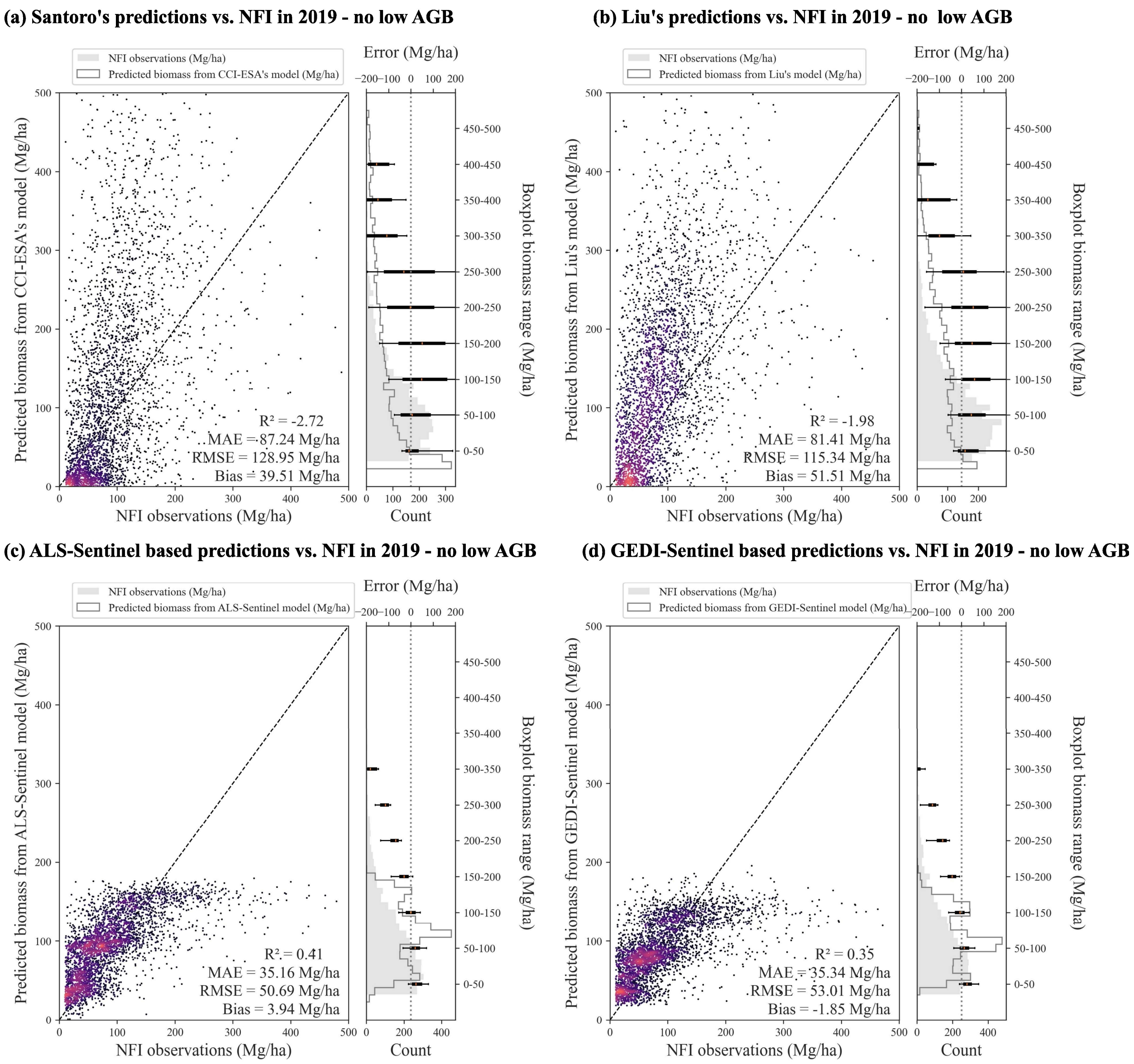

Supplementary figure 24 | The comparison of ALS and GEDI AGB predictions in 2019 with the products from Liu [9] and Santoro [10] based on the NFI AGB data without low values. Plot a is the scatter plot of predicted AGB of Santoro in 2019 versus the NFI recorded AGB without low values of 2019. Plot b is the scatter plot of predicted AGB of Liu in 2019 versus the NFI recorded AGB without low values of 2019. Plot c is the scatter plot of predicted AGB of our ALS-based RF AGB model in 2019 versus the NFI recorded AGB without low values of 2019. Plot d is the scatter plot of predicted AGB of our GEDI-based RF AGB model in 2019 versus the NFI recorded AGB without low values of 2019. The boxplots on the right side indicate the mean absolute errors in each AGB class.

Tables

Supplementary table 1 Downloading settings and access of training data

Products	Filters/Operations	Link to download the datasets	Size	Spatial coverage	Temporal coverage	Composting method
Sentinel-1	Mask out the data when value less than -30	• GEE code for data download: https://code.earthengine.google.com/8ec80583b3bcc7c1654bb83d657bf0a2<br>• Sentinel 1 2017: https://doi.org/10.5281/zenodo.14650832<br>• Sentinel 1 2018: https://doi.org/10.5281/zenodo.14650919<br>• Sentinel 1 2019: https://doi.org/10.5281/zenodo.14651053<br>• Sentinel 1 2020: https://doi.org/10.5281/zenodo.14651159<br>• Sentinel 1 2021: https://doi.org/10.5281/zenodo.14652535	2017: 17.42GB<br>2018: 18.05GB<br>2019: 18.42GB<br>2020: 16.85GB<br>2021: 16.94GB	Longitude (-9, 4) Latitude (36, 44)	2017.01.01 - 2021.12.31	Annual median
Sentinel-2	Removed the data when cloud cover is higher than 5% (based on QA60 band)	• GEE code for data download (including the cloud filtering code): https://code.earthengine.google.com/70a5ce97ccbc9d4c61bf08047999298f<br>• Sentinel 2 2017 p1: https://doi.org/10.5281/zenodo.14654157<br>• Sentinel 2 2017 p2: https://doi.org/10.5281/zenodo.14662104	2017 p1: 43.22GB<br>2017 p2: 49.59GB<br>2017 real color: 32.66GB		2017.01.01 - 2021.12.31	

		• Sentinel 2 2017 real color: https://doi.org/10.5281/zenodo.14652687<br>• Sentinel 2 2017 part 3 p1: https://doi.org/10.5281/zenodo.14665902<br>• Sentinel 2 2017 part 3 p2: https://doi.org/10.5281/zenodo.14666341<br>• Sentinel 2 2018 p1: https://doi.org/10.5281/zenodo.14662975<br>• Sentinel 2 2018 p2: https://doi.org/10.5281/zenodo.14663400<br>• Sentinel 2 2018 real color: https://doi.org/10.5281/zenodo.14652818<br>• Sentinel 2 2018 part 3 p1: https://doi.org/10.5281/zenodo.14666934<br>• Sentinel 2 2018 part 3 p2: https://doi.org/10.5281/zenodo.14667495<br>• Sentinel 2 2019 p1: https://doi.org/10.5281/zenodo.14663832<br>• Sentinel 2 2019 p2: https://doi.org/10.5281/zenodo.14664258<br>• Sentinel 2 2019 real color: https://doi.org/10.5281/zenodo.14652964<br>• Sentinel 2 2019 part 3 p1: https://doi.org/10.5281/zenodo.14668143	2017 part3 p1: 35.56GB<br><br>2017 part3 p2: 39.33GB<br><br>2018 p1: 42.49GB<br><br>2018 p2: 48.57GB<br><br>2018 real color: 30.24GB<br><br>2018 part3 p1: 35.97GB<br><br>2018 part3 p2: 39.45GB<br><br>2019 p1: 42.59GB<br><br>2019 p2: 48.42GB<br><br>2019 real color: 29.89GB			

		• Sentinel 2 2019 part 3 p2: https://doi.org/10.5281/zenodo.14668530<br>• Sentinel 2 2020 p1: https://doi.org/10.5281/zenodo.14664594<br>• Sentinel 2 2020 p2: https://doi.org/10.5281/zenodo.14664822<br>• Sentinel 2 2020 real color: https://doi.org/10.5281/zenodo.14653338<br>• Sentinel 2 2020 part 3 p1: https://doi.org/10.5281/zenodo.14669269<br>• Sentinel 2 2020 part 3 p2: https://doi.org/10.5281/zenodo.14669781<br>• Sentinel 2 2021 p1: https://doi.org/10.5281/zenodo.14665344<br>• Sentinel 2 2021 p2: https://doi.org/10.5281/zenodo.14665576<br>• Sentinel 2 2021 real color: https://doi.org/10.5281/zenodo.14653606<br>• Sentinel 2 2021 part 3 p1: https://doi.org/10.5281/zenodo.14670235<br>• Sentinel 2 2021 part 3 p2: https://doi.org/10.5281/zenodo.14670678	2019 part3 p1: 35.84GB<br>2019 part3 p2: 39.17GB<br>2020 p1: 43.08GB<br>2020 p2: 46.96GB<br>2020 real color: 30.25GB<br>2020 part3 p1: 36.32GB<br>2020 part3 p2: 39.13GB<br>2021 p1: 42.54GB<br>2021 p2: 48.16GB<br>2021 real color: 29.98GB			

			2021 part3 p1: 35.80GB<br><br>2021 part3 p2: 39.10GB			
GEDI canopy height	quality_flag = 1<br>Only powerful beams<br>degrade_flag = 0<br>sensitivity >= 0.98<br>0 < Rh98 < 50<br>energy_total >= 5000<br>solar_elevation < 0<br>num_detectedmodes > 0<br>slope.lte(10)<br>Remove the footprints when within 25m of the forest edges<br>When land use ("ESA/WorldCover/v200") is permanent water body and built-up, change value to 2.5m	• GEE code for data download: https://code.earthengine.google.com/7f9dc05850ecd689aa2c8fc1a49e4597<br><br>• GEDI footprint 2019-2021: https://doi.org/10.5281/zenodo.14650788	1.96GB		2019.03.25<br>-<br>2021.12.31	
GEDI tree coverage ratio	l2b_quality_flag = 1<br>Only powerful beams<br>degrade_flag = 0<br>sensitivity >= 0.98<br>solar_elevation < 0<br>slope.lte(10)<br>Remove the footprints when within 25m of the forest edges<br>When land use ("ESA/WorldCover/v200") is permanent water body and built-up, change value to 0	• GEE code for data download: https://code.earthengine.google.com/7b79f46e593e236957f0f638ff0ab17d<br><br>• GEDI tree coverage ratio footprint 2019-2021: https://doi.org/10.5281/zenodo.14712438	1.96GB	Longitude (-9, 4)<br>Latitude (36, 44)	2019.03.25<br>-<br>2021.12.31	

Original DSM and DTM		https://www.idee.es/csw-inspire-idee/srv/spa/catalog.search?#/metadata/spaignLIDAR_cob2		205,213 km^2 (35.18% of the Iberian Peninsula)	2017-2021	
Processed ALS data	ALS = DSM – DTM<br>Remove data when slope is greater than 50 °<br>Remove data when value is lower than 0.1m and higher than 45m	• ALS 2017: https://doi.org/10.5281/zenodo.14568145<br>• ALS 2018: https://doi.org/10.5281/zenodo.14568378<br>• ALS 2019: https://doi.org/10.5281/zenodo.14568468<br>• ALS 2020: https://doi.org/10.5281/zenodo.14570210<br>• ALS 2021: https://doi.org/10.5281/zenodo.14570264	2017: 32.19GB<br>2018: 33.92GB<br>2019: 44.54GB<br>2020: 5.52GB<br>2021: 7.46GB	Longitude (-9, 4) Latitude (36, 44)	2017-2021	

Supplementary table 2 | UNET model architecture in our study – Encoder, Bottleneck, Decoder

Step	Layer	Input Size	Output Size	Kernel/Operation	Channels
1	inc (DoubleConv)	H × W × n_channels	H × W × 64	3×3 Conv, ReLU (twice)	64
2	down1 (Down)	H × W × 64	H/2 × W/2 × 128	2×2 Max Pool + 3×3 Conv, ReLU (twice)	128
3	down2 (Down)	H/2 × W/2 × 128	H/4 × W/4 × 256	2×2 Max Pool + 3×3 Conv, ReLU (twice)	256
4	down3 (Down)	H/4 × W/4 × 256	H/8 × W/8 × 512	2×2 Max Pool + 3×3 Conv, ReLU (twice)	512
5	down4 (Down)	H/8 × W/8 × 512	H/16 × W/16 × 1024 (or 512 if bilinear)	2×2 Max Pool + 3×3 Conv, ReLU (twice)	512
6	down4 (Bottleneck)	H/16 × W/16 × 512	H/16 × W/16 × 512	3×3 Conv, ReLU (twice)	512
7	up1 (Up)	H/16 × W/16 × 512	H/8 × W/8 ×256	2×2 Up-conv, Skip Connection, 3×3 Conv, ReLU (twice)	256
8	up2 (Up)	H/8 × W/8 × 256	H/4 × W/4 × 128	2×2 Up-conv, Skip Connection, 3×3 Conv, ReLU (twice)	128
9	up3 (Up)	H/4 × W/4 × 128	H/2 × W/2 × 64	2×2 Up-conv, Skip Connection, 3×3 Conv, ReLU (twice)	64
10	up4 (Up)	H/2 × W/2 × 64	H × W × 64	2×2 Up-conv, Skip Connection, 3×3 Conv, ReLU (twice)	64

Supplementary table 3 | UNET model settings

Parameter	Value
Optimizer	Adam
Initial Learning Rate	0.01
Learning Rate Decay	Reduce by 0.9 every 10 loops if no improvement
Loss Function	Mean Absolute Error (MAE)

Ecological Insights from the datasets

Statistical analysis from ALS-Sentinel based models, as shown in **Supplementary figure 16a**, indicated that the mean canopy height in the forests of the Iberian Peninsula was 11.60 m in 2021. Specifically, in the Northern Iberian Peninsula, the mean forest canopy height was measured at 12.32 m. Conversely, in the Southern Iberian Peninsula, the forests exhibited a mean canopy height of 9.02 m. This data highlights that canopy heights in the forests of the Northern Iberian Peninsula are generally higher than those in the Southern Iberian Peninsula. **Supplementary figure 16b** further revealed that the mean forest AGB was 106.34 Mg/ha. In particular, the Northern Iberian Peninsula showed a mean forest AGB of 121.01 Mg/ha, while the forests in the Southern Iberian Peninsula had a mean AGB of 76.03 Mg/ha. It's important to note that the definition of forest in this analysis adheres to the GLC_FCS30D forest classification [13], and in this statistical analysis, only vegetation exceeding 5 m in height was included.

Compared to the data from 2017, there was an increase of 0.31 m in the mean canopy height within forest regions as detailed in **Supplementary figure 16**. Specifically, the Northern Iberian Peninsula saw a 0.39 m increase in mean canopy height, whereas the Southern Iberian Peninsula experienced a 0.03 m decrease. This variation suggests that disturbances and land-use changes had a more pronounced effect on the forests of the Southern Iberian Peninsula than those in the Northern Iberian Peninsula.

Supplementary figure 17a showed that between 2017 and 2021, 4.37-4.66% of forest areas (0.84-0.89 million hectares) experienced disturbances. Here the disturbance is defined only if there is a decrease in canopy height greater than 4 meters, which corresponds to the MAE of the GEDI-Sentinel based UNET model. Among these disturbances, the majority were small, affecting forest patches smaller than 1 hectare and accounting for 81.4% of the total. In contrast, large disturbances, affecting areas larger than 5 hectares, comprised 11% of the disturbances (**Supplementary figure 17b**).

As depicted **Supplementary figure 17a**, the ALS-Sentinel based model tends to report slightly higher levels of disturbance compared to the Sentinel-Sentinel based model. This difference can be attributed to the ALS-Sentinel based UNET's capability to identify individual trees, as illustrated in **Supplementary figure 18**, which benefits from the high spatial resolution and the inclusion of adjacent information in its training dataset. On the other hand, the GEDI data is characterized by larger gaps between laser footprints and significant spacing between tracks, impacting its disturbance detection performance [2]. These differences in data granularity led to the difference in disturbance detection performance for ALS-Sentinel based and GEDI-Sentinel based models. **Supplementary figure 19** illustrated the differences between ALS disturbance mask and GEDI disturbance mask. **Supplementary figure 20** explored how each model performs in high-mountain areas, highlighting that snow cover affects the predictions made by the GEDI-Sentinel based UNET model. Conversely, the ALS-Sentinel based UNET model is unaffected by snow cover, demonstrating its greater reliability for mapping in mountainous regions prone to snow.

Correction of NFI Data Using ALS Observations: Systematic Bias Adjustment and Disturbance Filtering

The adjustments included 2 parts, first part is the systematic bias, the second part is the potential disturbances.

Systematic Bias:

Identification of Systematic Bias:

Before using the NFI data for external validation, we compared the ALS-derived/observed canopy heights with the NFI observations at the same locations using a scatterplot. This comparison revealed a consistent pattern where the NFI observations were systematically higher than the ALS observations across the dataset (**Supplementary figure 21a**). This discrepancy is possibly due to the differences in measurement techniques for canopy height: NFI relies on manual measurements (e.g., hypsometer) on the maximum canopy height, which could introduce overestimations due to human error [12] (**Supplementary figure 22**). In the meanwhile, the ALS data used in this data have a relative low pulse density, with the range of pulse density of 0.5-14 pulses per square meter, which could potentially miss the highest part of the canopy and resulting in underestimation of maximum canopy heights.

Quantifying the Discrepancy:

To quantify the systematic bias, we calculated the root mean square error (RMSE) between the ALS observations and the NFI observations. We found that subtracting 1.5 meters from the NFI observations minimized the RMSE, indicating that this adjustment effectively aligned the NFI data with the ALS measurements (**Supplementary figure 21b**).

Rationale for the Correction:

The correction of NFI observations by 1.5 meters was applied to account for the systematic overestimation in manual measurements. Without this adjustment, as the NFI data were systematically higher than ALS observations, the NFI data would consistently overestimate tree heights compared to the ALS-derived predictions, leading to an unfair evaluation of the UNET model's performance.

By applying this correction, the validation process ensures that the ALS-trained model is evaluated against a more consistent reference dataset, thereby providing a fair assessment of the model's predictive accuracy.

Nevertheless, we also present the external validation results using the original, uncorrected NFI data (**Supplementary figure 23**). These results demonstrate that, although the predictive accuracy of both the ALS–Sentinel based and GEDI–Sentinel based UNET models slightly decreases with uncorrected NFI observations, their performance still exceeds that of global models from previous studies [5,6].

Potential disturbances:

When plotting the scatterplot of NFI observations and ALS observations, we found that for a very few points, we have a 0 value in ALS observations and a normal NFI observations at the same location, this is

likely due to the disturbances such as fires that occurred within the year of the NFI visit. And we simply removed those points.

AGB without low values

Since artificial zero values were introduced into the training and testing datasets, we excluded these data for model comparison. Accordingly, we provide an alternative version of the scatterplots based on NFI AGB without low values (**Supplementary Figure 24**). Compared to **Supplementary Figure 12**, the performance metrics decrease slightly but remain generally consistent—a reduction of approximately 0.02–0.03 in the coefficient of determination. This is because only a small fraction of zero values were added inside the dataset.

References

1. The Spanish Ministry of Transportation and Sustainable Mobility. LIDAR 2ª Cobertura (2015-2022) de España. https://www.idee.es/csw-inspire-idee/srv/spa/catalog.search?#/metadata/spaignLIDAR_cob2.
2. University of Maryland. GEDI. https://gedi.umd.edu/mission/technology/#:~:text=Relative%20Height%20(RH)%20metrics%20give,center%20of%20the%20ground%20return).
3. Alberdi, I., Cañellas, I. & Vallejo Bombín, R. The Spanish National Forest Inventory: history, development, challenges and perspectives. *Pesqui Florest Bras* **37**, 361 (2017).
4. Ronneberger, O., Fischer, P. & Brox, T. U-Net: Convolutional Networks for Biomedical Image Segmentation. in 234–241 (2015). doi:10.1007/978-3-319-24574-4_28.
5. Lang, N., Jetz, W., Schindler, K. & Wegner, J. D. A high-resolution canopy height model of the Earth. *Nat Ecol Evol* **7**, 1778–1789 (2023).
6. Potapov, P. *et al.* Mapping global forest canopy height through integration of GEDI and Landsat data. *Remote Sens Environ* **253**, 112165 (2021).
7. Takaku, J., Tadono, T., Doutsu, M., Ohgushi, F. & Kai, H. UPDATES OF 'AW3D30' ALOS GLOBAL DIGITAL SURFACE MODEL IN ANTARCTICA WITH OTHER OPEN ACCESS DATASETS. *The International Archives of the Photogrammetry, Remote Sensing and Spatial Information Sciences* **XLIII-B4-2021**, 401–408 (2021).
8. Duncanson, L. *et al.* Aboveground biomass density models for NASA's Global Ecosystem Dynamics Investigation (GEDI) lidar mission. *Remote Sens Environ* **270**, 112845 (2022).
9. Liu, S. *et al.* The overlooked contribution of trees outside forests to tree cover and woody biomass across Europe. *Sci Adv* **9**, (2023).
10. Santoro, M. & Cartus, O. ESA Biomass Climate Change Initiative (Biomass_cci): Global datasets of forest above-ground biomass for the years 2010, 2015, 2016, 2017, 2018, 2019, 2020 and 2021, v5. . *NERC EDS Centre for Environmental Data Analysis* (2024).
11. Guerra-Hernández, J., Pereira, J. M. C., Stovall, A. & Pascual, A. Impact of fire severity on forest structure and biomass stocks using NASA GEDI data. Insights from the 2020 and 2021 wildfire season in Spain and Portugal. *Science of Remote Sensing* **9**, 100134 (2024).
12. Villaescusa, R. & Díaz, R. *Segundo Inventario Forestal Nacional (1986–1996)*. (Spain: Ministerio de Medio Ambiente, ICONA, Madrid, 1998).
13. Zhang, X. *et al.* GLC_FCS30D: the first global 30 m land-cover dynamics monitoring product with a fine classification system for the period from 1985 to 2022 generated using dense-time-series Landsat imagery and the continuous change-detection method. *Earth Syst Sci Data* **16**, 1353–1381 (2024).
